# The NALCN channel complex is necessary for stabilizing sleep in *Caenorhabditis elegans*

**DOI:** 10.1093/g3journal/jkag112

**Published:** 2026-05-05

**Authors:** Takahiro Kamijo, Shinichi Miyazaki, Yu Hayashi

**Affiliations:** Department of Biological Sciences, Graduate School of Science, The University of Tokyo, Bunkyo-ku, Tokyo 113-0033, Japan; Department of Biological Sciences, Graduate School of Science, The University of Tokyo, Bunkyo-ku, Tokyo 113-0033, Japan; International Institute for Integrative Sleep Medicine (WPI-IIIS), Tsukuba Institute for Advanced Research (TIAR), University of Tsukuba, Tsukuba, Ibaraki 305-8575, Japan; Department of Biological Sciences, Graduate School of Science, The University of Tokyo, Bunkyo-ku, Tokyo 113-0033, Japan; International Institute for Integrative Sleep Medicine (WPI-IIIS), Tsukuba Institute for Advanced Research (TIAR), University of Tsukuba, Tsukuba, Ibaraki 305-8575, Japan

**Keywords:** *Caenorhabditis elegans*, NALCN, UNC-79, UNC-80, sleep stabilization, early period of sleep

## Abstract

Sleep is a fundamental physiological state that is widely conserved across diverse animal species. *Caenorhabditis elegans* exhibits developmentally timed sleep (DTS) and stress-induced sleep (SIS), which meet the behavioral definition of sleep and are regulated by molecular mechanisms shared with other animals. Here, we studied the sleep-regulating functions of UNC-80, which forms a complex with NCA-1 and NCA-2 (hereafter collectively referred to as NCA), and UNC-79. We found that the *unc-80* loss-of-function (lf) mutants experienced lower quiescence and a more fragmented and longer DTS than the wild type. The *unc-80(lf)* mutants also showed lower quiescence during SIS immediately after heat stress, and fragmented SIS. After that, however, quiescence remained higher for a longer time, resulting in a higher total quiescence than observed in the wild type. Similar sleep defects were observed in *nca(lf)* and *unc-79(lf)* mutants, indicating that these genes have common functions in sleep regulation. Deprivation of SIS immediately after heat stress in the wild type resulted in a sleep pattern similar to that of the *unc-80(lf)* mutants, suggesting that sleep immediately after stress might be essential and thus is under strong homeostatic regulation and that the prolonged DTS and SIS of the *unc-80(lf)* mutants might be rebound sleep. Our findings elucidated that NCA, UNC-79, and UNC-80 are necessary for stabilizing sleep and suggest the importance of the early period of sleep.

## Introduction

Sleep is a widely conserved behavioral state across the animal kingdom. Behavioral definitions of sleep include rapid reversibility to arousal, stereotypic postures, elevated arousal threshold, and homeostatic regulation (Campbell and Tobler 1984; Rial et al. 2010). Prolonged sleep deprivation can be fatal, indicating that sleep is essential for survival (Rechtschaffen et al. 1983; Shaw et al. 2002; Driver et al. 2013). Sleep fragmentation leads to more daytime sleepiness and impairs mood and attention even when the sleep amount is not decreased (Martin et al. 1996). These studies suggest the importance of sleep stabilization and the likelihood that some mechanisms exist to maintain it. The molecular mechanisms of sleep stabilization, however, remain largely unknown. Humans enter a particularly deep sleep called slow-wave sleep shortly after sleep onset, during which the immune system is activated (Besedovsky et al. 2012) and growth hormone is released (VanCauter and Plat 1996). Loss of slow-wave sleep is associated with some diseases like dementia (Himali et al. 2023) and hypertension (Javaheri et al. 2017), suggesting that sleep immediately after sleep onset might be especially important for human health. Whether this particular importance of early sleep exists widely among animal species, however, remains unclear.

The nematode *Caenorhabditis elegans* undergoes 4 molts before reaching adulthood. Before each molt, nematodes show a reversible quiescent state called developmentally timed sleep (DTS) (Singh and Sulston 1978). During DTS, *C. elegans* exhibits an elevated arousal threshold, and DTS is indicated to be homeostatically regulated (Raizen et al. 2008). These characteristics are included in the behavioral definition of sleep. *C. elegans* also exhibits a quiescent state after exposure to stressors including heat, pore-forming toxins (Hill et al. 2014), and ultraviolet light (DeBardeleben et al. 2017). This state is called stress-induced sleep (SIS), which meets the behavioral definitions of sleep (Hill et al. 2014; Soto et al. 2019). Many molecules and molecular pathways that regulate DTS and SIS also regulate sleep in a wide range of animal species, including mammals. Such molecules include the AP2 transcription factor APTF-1 (Turek et al. 2013; Kucherenko et al. 2016; Hu et al. 2020; Nakai et al. 2020), the salt-inducible kinase SIK3 (Funato et al. 2016), the histone deacetylase HDAC4 (Kim et al. 2022), protein kinase G (Raizen et al. 2008; Langmesser et al. 2009), epidermal growth factor signaling (Kushikata et al. 1998; Kramer et al. 2001; Foltenyi et al. 2007; Van Buskirk and Sternberg 2007), cyclic adenosine monophosphate signaling pathway (Hendricks et al. 2001; Graves et al. 2003; Raizen et al. 2008; Singh et al. 2014), and the dopaminergic pathway (Singh et al. 2014; Kashiwagi et al. 2021). Thus, *C. elegans* sleep may be homologous to sleep in many other animals, which suggests that studying the sleep of *C. elegans* can lead to a universal understanding of sleep.

NALCN (Na+ leak channel, nonselective) is a voltage-independent sodium leakage channel that regulates the neuronal resting membrane potential (Ren 2011). NALCN forms a complex with 2 auxiliary subunits, UNC79 and UNC80 (Lu et al. 2010; Kschonsak et al. 2022). NALCN, UNC79, and UNC80 are widely conserved in the animal kingdom (Humphrey et al. 2007; Jospin et al. 2007; Lu et al. 2010). In humans, mutations in *NALCN*, *UNC79*, and *UNC80* cause hypotonia, psychomotor retardation, and characteristic facial features (Köroglu et al. 2013; Flourakis et al. 2015; Gal et al. 2016; Perez et al. 2016; Stray-Pedersen et al. 2016; He et al. 2018; Bayat et al. 2023), called NALCN channelopathies (Bramswig et al. 2018). *NALCN*, *UNC79,* and *UNC80* have a variety of functions, and many functions are conserved among a wide range of animals, including circadian rhythm formation (Nash et al. 2002; Lear et al. 2013; Flourakis et al. 2015), locomotion (Krishnan and Nash 1990; Humphrey et al. 2007; Jospin et al. 2007; Yeh et al. 2008), and sensitivity to alcohol (Speca et al. 2010) and halothane (Humphrey et al. 2007). In addition, *NALCN*, *UNC79*, and *UNC80* mutations disrupt sleep in many animals, including humans. In humans, sleep disturbances are reported in patients with mutations in *NALCN* (Angius et al. 2018) and *UNC80* (Stray-Pedersen et al. 2016; He et al. 2018). *Dreamless* mutant mice, which have gain-of-function mutations in *Nalcn*, have reduced rapid eye movement sleep (Funato et al. 2016). Fruit flies with the *unc79* loss-of-function mutation have increased sleep (Murakami et al. 2021). In *C. elegans*, there are 2 homologs of *Nalcn*, *nca-1* and *nca-2*, which work redundantly (Humphrey et al. 2007; Yeh et al. 2008). The *nca-2;nca-1(lf)* double mutants (hereafter, *nca(lf)* mutant) and the *unc-79(lf)* and *unc-80(lf)* mutants have higher arousal thresholds than the wild type (N2 strain) during DTS sleep bouts, and *unc-79(lf)* mutants exhibit an increased amount of sleep (Huang et al. 2018). The detailed sleep patterns of these mutant animals have not been elucidated, however, and why mutations in NALCN or its auxiliary subunits lead to sleep disruption remains largely unknown.

A previous study reported that *unc-80(lf)* mutants have normal sleep amounts during DTS (Huang et al. 2018). However, under different experimental conditions and with higher imaging frequency, we found that *unc-80(lf)* mutants sleep more than wild type and have a characteristic sleep pattern. We were interested in determining the precise functions of *unc-80* and whether *nca*, *unc-79*, and *unc-80* have a common function. Therefore, We examined the sleep of *nca(lf)*, *unc-79(lf),* and *unc-80(lf)* mutants in more detail. We found that these mutants exhibit fragmented sleep and reduced quiescent state fractions during both the early period of DTS and the early period of SIS, indicating an unstable sleep pattern during sleep immediately after sleep onset. However, they showed prolonged duration of DTS and SIS, which might be a result of homeostatic responses to the disturbance of the early period of sleep. These findings suggest that the early period of sleep is especially important. Together, our findings reveal that *nca*, *unc-79*, and *unc-80* are necessary for sleep stabilization and provide new insights into the importance of the early period of sleep.

## Methods

### Strains

*C*. *elegans* were cultured at 20 °C on nematode growth medium (NGM) agar plates containing streptomycin. The plates were seeded with *Escherichia coli*  OP50-1 as a food source (Brenner 1974).

Strains used in this study are listed in the Reagent Table. For the rescue experiment, ZM4624 was used as a control to ensure genetic background consistency with ZM7212. Among ZM7212, RFP+ worms were used as the rescue group, while RFP- worms were used as the *nca(lf)* mutant group.

### DTS measurements

DTS during the transition from L4 to adulthood was captured using the method described previously (Singh et al. 2011; Chen et al. 2023; Kawano et al. 2023) with some modifications. A polydimethylsiloxane (PDMS) chamber with an 8 × 6 array of wells (1 mm in diameter, 0.5 mm deep) was adhered to the center of a slide glass. OP50-1 liquid culture with A600 absorption of 1.0∼1.5 was resuspended in liquid NGM at a 1:19 ratio and applied to each well of the PDMS chamber before loading the worms. Mid-L4 stage worms were judged by the vulva morphology and presence of pharyngeal pumping and loaded into each well. Then, the PDMS chamber was covered with a plastic sheet and placed on a stage made of LEGO blocks (LEGO). The worms were imaged by our rapid *C. elegans* motion imaging (Remi) system (Singh et al. 2011; Chen et al. 2023; Kawano et al. 2023) composed of a Raspberry Pi coupled to Raspberry Pi Camera Module 2 NOIR (http://www.raspberrypi.org/) and dark-field illumination with a red LED from the side of the camera. The whole stage was covered with an empty box to shade the room light during image acquisition. Image acquisition and analysis were performed with custom-made Python scripts using different parameters from the previous studies (Singh et al. 2011; Chen et al. 2023; Kawano et al. 2023) to detect DTS more accurately (https://github.com/TakahiroKamijo/ImageSubt_analysis.git). Images were obtained every 2 s for 10 h. Movements of the worms were detected using an image subtraction method (Singh et al. 2011). The pixel values were subtracted between consecutive images, and then the subtracted images were subjected to a median filter, normalization, and binarization. If the sum of changed pixels from the region of interest was >1% of the body size of the worm, the animal was judged as active and, if not, quiescent. Then, the fraction of quiescence (FOQ) was calculated in a 10-min rolling window. DTS was defined as the period during which the FOQ remained >0.05 for more than 1 h. All comparisons were performed by simultaneously imaging the indicated strains in the same PDMS chamber; this procedure was repeated across multiple independent imaging sessions.

### Developmental speed measurements

Image acquisition and processing were performed in the same way as described above except that images were captured using a fluorescence stereo microscope (M205 FA, Leica), camera (MC120 HD, Leica), and Micro-Manager 2.0 (Edelstein et al. 2014). Mid-L3 stage worms were judged by the vulva morphology and presence of pharyngeal pumping and were imaged for 24 h to calculate the interval between the start of L3 DTS and the start of L4 DTS. All comparisons were performed by simultaneously imaging the indicated strains in the same PDMS chamber; this procedure was repeated across multiple independent imaging sessions.

### SIS measurements

Image acquisition and processing were performed for the SIS measurements in the same way as for the DTS measurements. For the SIS measurements, young adult animals with fully developed vulvae and no embryos in the uterus were selected. Young adult animals were loaded into the PDMS chamber and incubated at 37 °C or 40 °C incubator for 20 min. Immediately after heat stress exposure, the animals were imaged for 6 h (37 °C) or 12 h (40 °C). Time-point zero represents the end of heat stress exposure. All comparisons were performed by simultaneously imaging the indicated strains in the same PDMS chamber; this procedure was repeated across multiple independent imaging sessions.

### Arousal threshold tests

For the arousal threshold test, young adult animals were selected and transferred to NGM agar plates, with fewer than 15 animals per plate. The plates were sealed with parafilm and submerged in the water bath at 37 °C for 20 min. After heat stress exposure, the plates were left in a 20 °C incubator for 20 min with the lids off and then for 1 min on the microscope stage before image acquisition. Images were captured using a fluorescence stereo microscope (M205 FA, Leica), camera (MC120 HD, Leica), and Micro-Manager 2.0 (Edelstein et al. 2014) every 250 ms for 4 min. Animals were illuminated with a blue LED ring light (LDR2-90BL2, CCS Inc.) with a wavelength of 470 nm and intensity of 0.18 mW/mm^2^ for the last 2 min of image acquisition. To analyze the images, we used the custom-made Python script (https://github.com/TakahiroKamijo/worm_tracking.git), which can track multiple animals at the same time. The background images of agar plates were subtracted from the original images, and the subtracted images were binarized. In binarized images, contours with an area greater than a threshold are detected as worms and surrounded by bounding boxes. The travel distance of the center of each bounding box during image acquisition was calculated. If the travel distance during the first 2 min of image acquisition exceeded a threshold (10 pixels), the animal was judged as awake before blue light exposure and removed from the following analysis. If an animal judged as asleep before blue light exposure moved longer than the threshold (10 pixels) during blue light exposure, then the animal was considered to respond to the blue light.

### SIS deprivation

Young adult animals were exposed to heat stress at 37 °C for 20 min in the same way as for the SIS measurement described above. Then, images were acquired using a fluorescence stereo microscope (M205 FA, Leica), camera (MC120 HD, Leica), and Micro-Manager 2.0 (Edelstein et al. 2014) every 2 s for 6 h. N2 animals were illuminated with a blue LED ring light (LDR2-90BL2, CCS Inc) for the first 30 min of image acquisition. Control N2 animals and *unc-80(lf)* mutants were not exposed to blue light. Acquired images were analyzed in the same way as for the SIS measurement described above. In this experiment, side-by-side comparison was not feasible because blue-light stimulation could not be reliably confined to individual wells. Therefore, data for each experimental condition was collected separately in independent recording sessions.

### Heat stress survival

The survival assay after exposure to heat stress was performed as described previously (Hill et al. 2014) with some modifications. Twelve young adult animals were transferred to each NGM plate, and the plates were immediately sealed with parafilm and floated on the water at 40 °C for 20 min. Immediately after exposure to heat stress, the water and parafilm on the plate were removed. The animals were incubated at 20 °C, and their survival was checked daily. Animals that did not respond to mechanical stimuli such as plate tapping or nose touching using a platinum wire were considered dead. Animals that were dried out on the wall of the plates, not found, showed vulval expulsion or a “bag of worms phenotype were excluded from the analysis. The survival assay was terminated at day 40 or when all worms were dead or censored.

### Statistical analysis

Statistical analysis was carried out using R (4.3.1) with ggsignif (0.6.4), multcomp (1.4 to 25), survival (3.8 to 3), and anovakun (4.8.9) packages. The statistical tests used are described in each figure legend. Statistical differences at *P* < 0.05 were considered significant.

## Results

### *unc-80* is required for stabilizing DTS

**Fig. 1.**
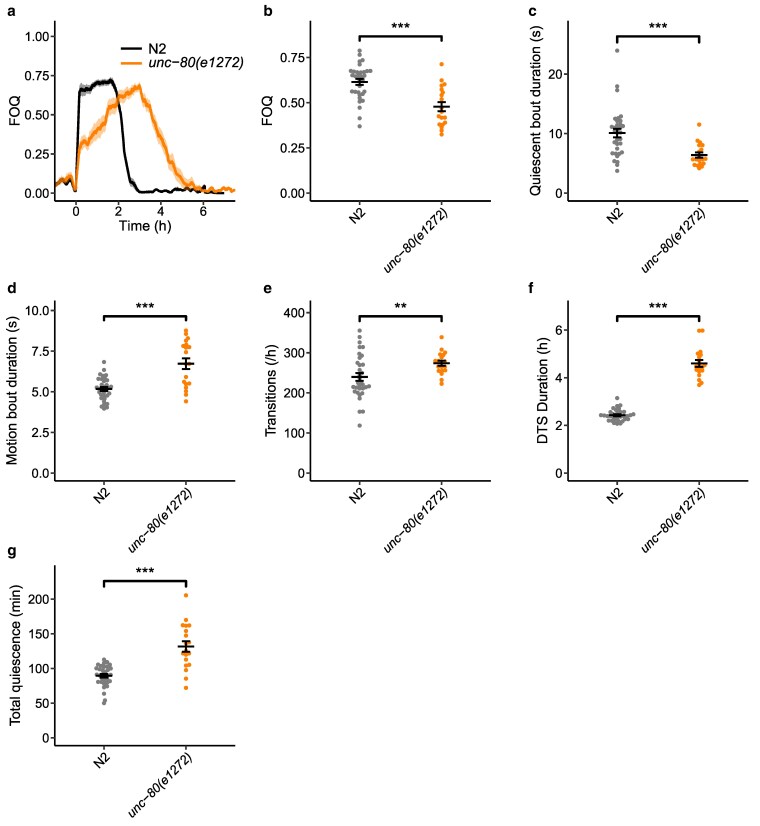
DTS is disrupted in *unc-80(lf)* mutants. a to e) Temporal pattern of FOQ (a) and FOQ (b), mean duration of quiescent bouts (c), mean duration of motion bouts (d), and transitions between quiescent and motion bouts per hour (e) during DTS for N2 (*n* = 33) and *unc-80(e1272)* (*n* = 19). f) Duration of DTS for N2 (*n* = 33) and *unc-80(e1272)* (*n* = 19). g) Total quiescent state amount during DTS for N2 (*n* = 33) and *unc-80(e1272)* (*n* = 19). In (a), the colored lines represent the mean FOQ and shadows around the lines represent the standard error (SE). In (b to g), each dot represents an individual animal, and bars represent mean ± SE. Welch's *t* test was performed. **P < 0.01; ***P < 0.001.

First, we examined DTS of *unc-80(e1272)* mutants, loss-of-function *unc-80* mutants, during L4 to adult transition. In N2, the FOQ sharply increased and decreased at the onset and end of DTS, respectively (Fig. 1a). By contrast, in the *unc-80(lf)* mutants, the FOQ increase at the onset of DTS appeared less sharp, and the FOQ thereafter gradually increased until it reached a peak level comparable to N2, then gradually decreased (Fig. 1a). The average FOQ during DTS was lower compared with N2 (Fig. 1b). The reduced average FOQ could be explained by a shortened mean quiescent bout duration and a prolonged mean motion bout duration (Fig. 1, c and d). In addition, transitions between quiescent and motion bouts were more frequent (Fig. 1e), indicating that the DTS of *unc-80(lf)* mutants was fragmented. On the other hand, the DTS duration was longer in the *unc-80(lf)* mutants than in N2 (Fig. 1f). As a result, the *unc-80(lf)* mutants had higher total quiescence during DTS than N2 (Fig. 1g). The interval between L3 and L4 DTS was not significantly different between N2 and *unc-80(lf)* mutants (Supplementary Fig. 1a in File 1). Therefore, the extension of DTS duration is not due to a general reduction of developmental speed.

*unc-80(lf)* mutants become paralyzed in liquid (Pierce-Shimomura et al. 2008), and this may affect the FOQ regardless of DTS considering that our measurement was conducted in a liquid medium. We thus examined the FOQ of animals out of DTS and found no significant difference between N2 and the *unc-80(lf)* mutants (Supplementary Fig. 1, b and c in File 1), indicating that the defect in the quiescent state of the *unc-80(lf)* mutants during DTS was not a result of paralysis in the liquid.

### *unc-80* is required for stabilizing sleep immediately after heat stress

**Fig. 2.**
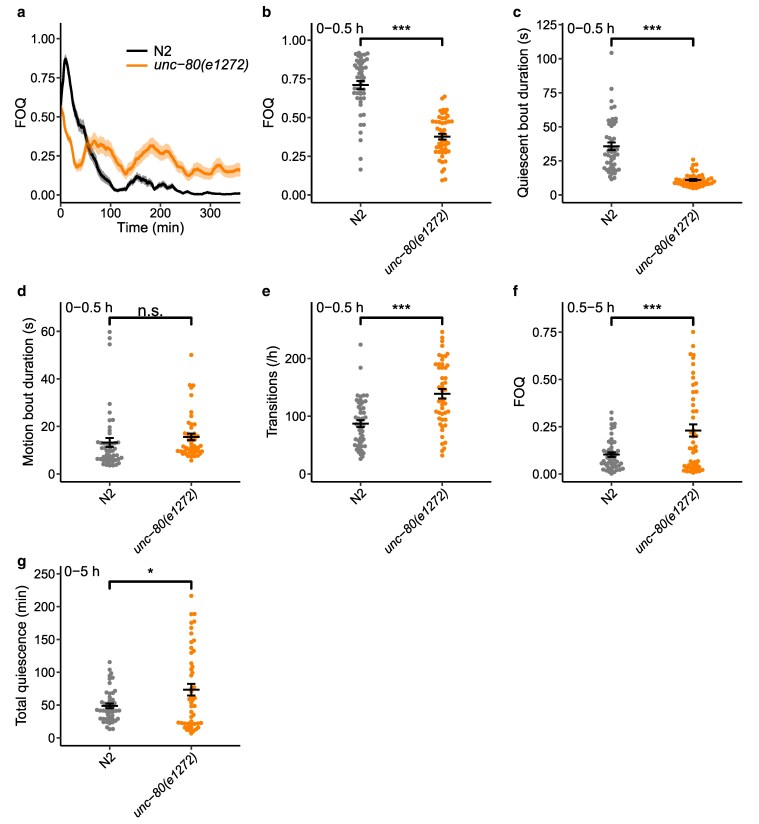
SIS is disrupted in *unc-80(lf)* mutants. a to g) Temporal pattern of FOQ (a), FOQ 0 to 0.5 h (b), mean duration of quiescent bouts 0 to 0.5 h (c), mean duration of motion bouts 0 to 0.5 h (d), transitions between quiescent and motion bouts per hour 0 to 0.5 h (e), FOQ 0.5 to 5 h (f), and total amount of quiescence 0 to 5 h (g) after heat stress exposure at 37 °C for 20 min for N2 (*n* = 48) and *unc-80(e1272)* (*n* = 48). In (a), the colored lines represent the mean FOQ and shadows around the lines represent SE. In (b to g), each dot represents an individual animal, and bars represent mean ± SE. Welch's *t* test was performed. *P < 0.05; ***P < 0.001; n.s., not significant.

Next, we examined SIS in *unc-80(lf)* mutants after heat stress by exposure to 37 °C for 20 min. In N2, the FOQ peaked at 10∼20 min and then decreased until about 100 min after heat stress and thereafter remained low (Fig. 2a). By contrast, the FOQ of the *unc-80(lf)* mutants appeared to drop immediately after heat stress (Fig. 2a), and statistical analysis revealed that the FOQ until 30 min after heat stress was lower than that of N2 (Fig. 2b). During this time, the mean quiescent bout duration was shorter, whereas the mean motion bout duration was not significantly different from N2 (Fig. 2, c and d). *unc-80(lf)* mutants showed more frequent transitions between quiescent and motion bouts (Fig. 2e), indicating that their sleep was fragmented similarly to DTS. Thereafter, however, the FOQ of the *unc-80(lf)* mutants appeared to increase and remain higher for at least several hours compared with N2 (Fig. 2a). Actually, the FOQ during 0.5 to 5 h after heat stress was higher (Fig. 2f), resulting in a higher total amount of quiescence during 0 to 5 h after heat stress than in N2 (Fig. 2g). On the other hand, there was no significant difference in the baseline FOQ in the absence of heat stress (Supplementary Fig. 2, a and b in File 1), indicating that the mutants do not have a general increase in quiescence that could confound their SIS phenotype. The abnormal sleep pattern during SIS is similar to that during DTS, which lasted longer and thus resulted in a higher total amount of quiescence compared with that of N2. These experiments suggest that *unc-80(lf)* mutants are unable to establish stable sleep and experience prolonged sleep.

### Similar DTS and SIS phenotypes are observed in strains carrying different *unc-80* loss-of-function mutant alleles

The *unc-80(e1272)* mutant is a loss-of-function mutant created by ethyl methanesulfonate mutagenesis. Therefore, it might contain additional mutations, and it is possible that mutations other than *unc-80(e1272)* affected the DTS and SIS in the *unc-80 (e1272*) mutants. Therefore, we examined the sleep of the *unc-80(e1069)* mutant, another loss-of-function mutant allele strain. The *unc-80(e1069)* mutants showed similar DTS features to *unc-80(e1272)* mutants, such as a lower FOQ during DTS and longer DTS duration, resulting in a similar FOQ trace to that of *unc-80(e1272)* mutants (Supplementary Fig. 3 in File 1). We next examined the SIS of *unc-80(e1069)* mutants. We found that *unc-80(e1069)* mutants also exhibited similar SIS features to *unc-80(e1272)* mutants (Supplementary Fig. 4 in File 1). These results indicate that the DTS and SIS defects in *unc-80* mutants are caused by *unc-80* loss-of-function mutations.

### Analyses of *nca* and *unc-79* mutants suggest common functions in sleep regulation among NCA, UNC-79, and UNC-80

**Fig. 3.**
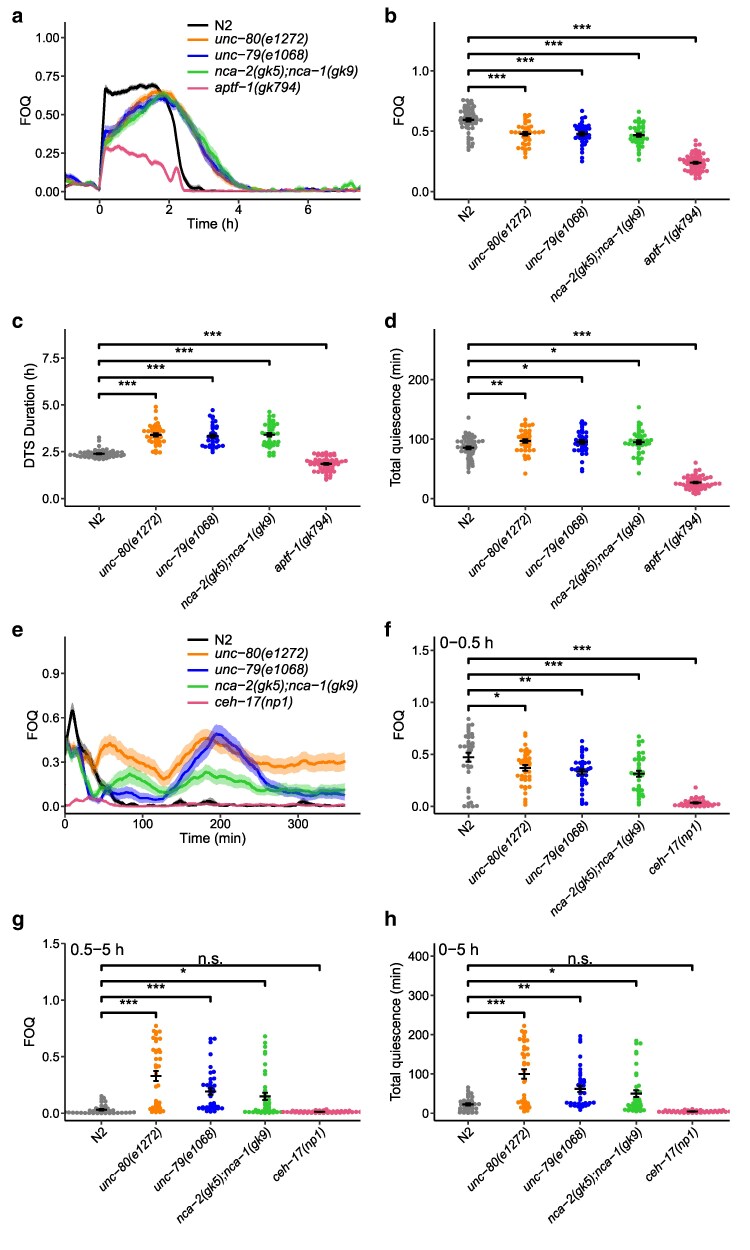
*unc-79(e1068)* mutants and *nca-2(gk5); nca-1(gk9)* double mutants have DTS and SIS patterns similar to *unc-80(e1272)*. (a) and (b) Temporal pattern of FOQ (a) and FOQ (b) during DTS for N2 (*n* = 58), *unc-80(e1272)* (*n* = 40), *unc-79(e1068)* (*n* = 37), *nca-2(gk5); nca-1(gk9)* (*n* = 42), and *aptf-1(gk794)* (*n* = 63). c) Duration of DTS for N2 (*n* = 58), *unc-80(e1272)* (*n* = 40), *unc-79(e1068)* (*n* = 37), *nca-2(gk5); nca-1(gk9)* (*n* = 42), and *aptf-1(gk794)* (*n* = 63). d) Total amount of quiescence during DTS for N2 (*n* = 58), *unc-80(e1272)* (*n* = 40), *unc-79(e1068)* (*n* = 37), *nca-2(gk5); nca-1(gk9)* (*n* = 42), and *aptf-1(gk794)* (*n* = 63). e to h) Temporal pattern of FOQ (e), FOQ 0 to 0.5 h (f), FOQ 0.5 to 5 h (g), and total amount of quiescence 0 to 5 h (h) after heat stress exposure at 37 °C for 20 min for N2 (*n* = 39), *unc-80(e1272)* (*n* = 39), *unc-79(e1068)* (*n* = 39), *nca-2(gk5); nca-1(gk9)* (*n* = 39), and *ceh-17(np1)* (*n* = 39). In (a) and (e), the colored lines represent the mean FOQ and shadows around the lines represent SE. In (b to d) and (f to h), each dot represents an individual animal, and bars represent mean ± SE. Multiple comparisons were performed using Dunnett's test with N2 as a control. *P < 0.05, **P < 0.01; ***P < 0.001; n.s., not significant.

To elucidate whether *nca(lf)* and *unc-79(lf)* mutants phenocopy the sleep defects observed in *unc-80(lf)* mutants, we examined sleep in *nca(lf)* and *unc-79(lf)* mutants. *nca(lf)* and *unc-79(lf)* mutants showed similar FOQ trends to *unc-80(lf)* mutants (Fig. 3a). We found that they had a lower FOQ, longer DTS duration, fragmented sleep, and a higher total amount of quiescence than N2 (Fig. 3, b to d and Supplementary Fig. 5 in File 1). On the other hand, *aptf-1(lf)* mutants, which exhibit largely reduced quiescence during DTS (Turek et al. 2013), had a lower FOQ, but showed a normal DTS duration and a lower total amount of quiescence than N2 (Fig. 3, b to d). These results suggested that *nca*, *unc-79*, and *unc-80* share a common function in the regulation of DTS and that they regulate DTS in a different manner than *aptf-1*.

Next, we examined their SIS. *nca(lf)* and *unc-79 (lf)* mutants showed a lower FOQ and sleep fragmentation for 30 min immediately after heat stress (Fig. 3, e and f and Supplementary Fig. 6 in File 1). On the other hand, their FOQ remained higher for 0.5∼5 h after heat stress, resulting in a higher total amount of quiescence (Fig. 3, g to h). These features are common to *unc-80(lf)* mutants, suggesting that *nca*, *unc-79*, and *unc-80* share a common function in the regulation of SIS. The apparent differences in the SIS patterns among these mutants (Fig. 3e), however, suggest that they may not have completely identical functions. In addition, *ceh-17(lf)* mutants, which have decreased quiescence after stress exposure (Hill et al. 2014), have a lower FOQ until 30 min after heat stress, and the FOQ remained lower thereafter (Fig. 3, e to g). This suggests that *nca*, *unc-79*, and *unc-80* regulate SIS in a different manner than *ceh-17*.

### *unc-80* loss-of-function mutants have normal arousal threshold during fragmented SIS

One of the behavioral definitions of sleep is an elevated arousal threshold (Campbell and Tobler 1984; Rial et al. 2010). *nca(lf)*, *unc-79(lf)*, and *unc-80(lf)* mutants had a lower FOQ and fragmented sleep for 30 min immediately after heat stress. Thus, we hypothesized that *nca(lf)*, *unc-79(lf)*, and *unc-80(lf)* mutants have a lower arousal threshold and are more responsive to external stimuli during sleep, which may lead to sleep disruption. Therefore, we examined the responsiveness of *unc-80(lf)* mutants to blue light, which is a noxious stimulus for *C. elegans*, 30 min after heat stress. The percentage of sleeping animals that responded to blue light was not different between *unc-80(lf)* mutants and N2 (Table 1). This result suggested that the arousal threshold in *unc-80(lf)* mutants is not defective during SIS and their sleep disruption is thus caused by other mechanisms.

**Table 1.** *unc-80 (e1272)* mutants have normal arousal threshold.

	% Respond	*N*
N2	45.5 ± 6.7	55
*unc-80(e1272)*	47.7 ± 6.2 ^n.s^	65

Percentage of animals that responded to blue light 30 min after heat stress for N2 and *unc-80(e1272)*. Fisher's exact test was performed. n.s., not significant.

### Panneuronal expression of *nca-1* almost completely rescues the DTS phenotype

**Fig. 4.**
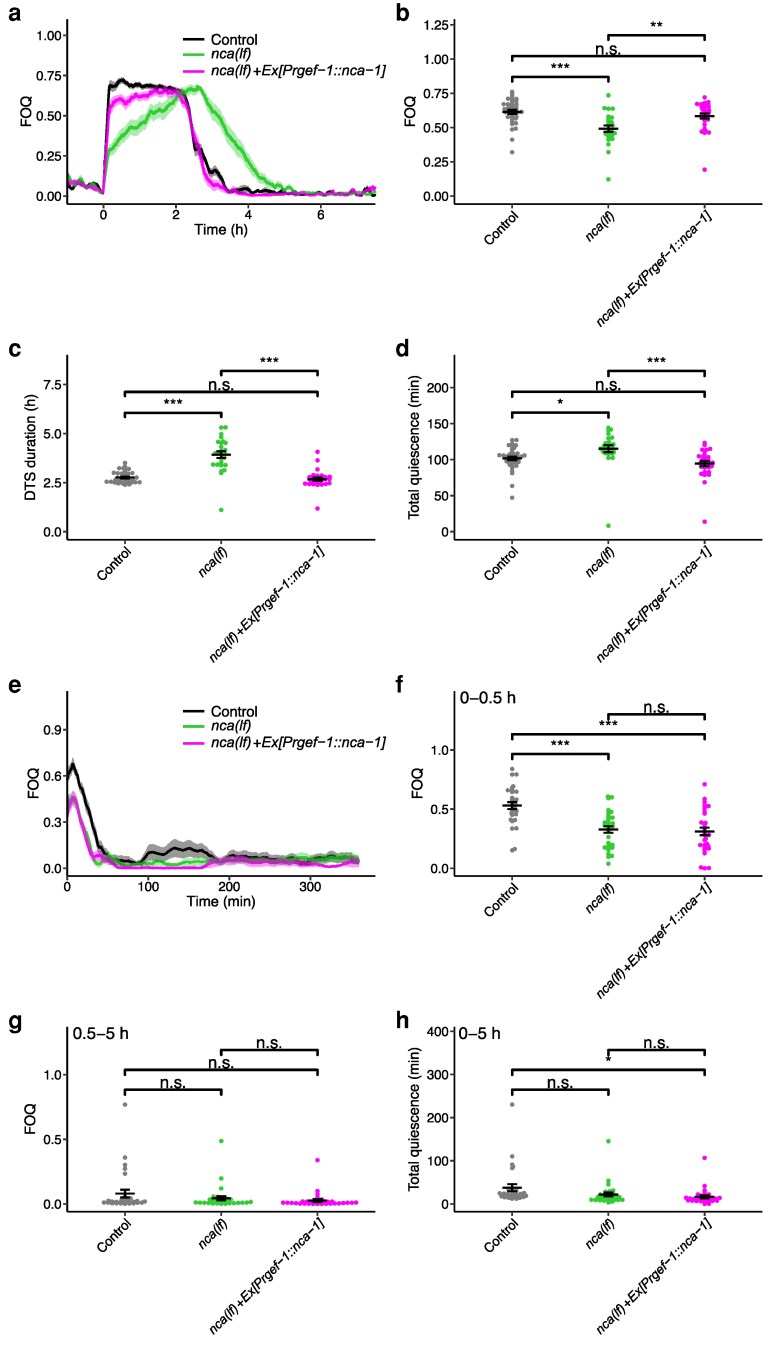
Panneuronal expression of *nca-1* rescues the DTS defects but not the SIS defects of the *nca-2(gk5); nca-1(gk9)* double mutants. (a) and (b) Temporal pattern of FOQ (a) and FOQ (b) during DTS for control (*Pglr-1::ChR2::YFP*) (*n* = 38), *nca-2(gk5); nca-1(gk9); Pglr-1::ChR2::YFP* (*n* = 26), and *nca-2(gk5); nca-1(gk9); Pglr-1::ChR2::YFP; Prgef-1::nca-1::GFP* (*n* = 30). c) Duration of DTS for control (*Pglr-1::ChR2::YFP*) (*n* = 38), *nca-2(gk5); nca-1(gk9); Pglr-1::ChR2::YFP* (*n* = 26), and *nca-2(gk5); nca-1(gk9); Pglr-1::ChR2::YFP; Prgef-1::nca-1::GFP* (*n* = 30). d) Total amount of quiescence during DTS for control (*Pglr-1::ChR2::YFP*) (*n* = 38), *nca-2(gk5); nca-1(gk9); Pglr-1::ChR2::YFP* (*n* = 26), and *nca-2(gk5); nca-1(gk9); Pglr-1::ChR2::YFP; Prgef-1::nca-1::GFP* (*n* = 30). e to h) Temporal pattern of FOQ (e), FOQ 0 to 0.5 h (f), FOQ 0.5 to 5 h (g) and total amount of quiescence 0 to 5 h (h) after heat stress exposure at 37 °C for 20 min for control (*Pglr-1::ChR2::YFP*) (*n* = 30), *nca-2(gk5); nca-1(gk9); Pglr-1::ChR2::YFP* (*n* = 32), and *nca-2(gk5); nca-1(gk9); Pglr-1::ChR2::YFP; Prgef-1::nca-1::GFP* (*n* = 32). In (a) and (e), the colored lines represent the mean FOQ and shadows around the lines represent SE. In (b to d) and (f to h), each dot represents an individual animal, and bars represent mean ± SE. Multiple comparisons were performed using Tukey–Kramer test. *P < 0.05; **P < 0.01; ***P < 0.001; n.s., not significant.

*nca-1, nca-2*, *unc-79*, and *unc-80* are widely expressed in the nervous system (Jospin et al. 2007; Yeh et al. 2008). Therefore, to determine whether the NALCN channel complex functions in the nervous system for DTS and SIS regulation, we measured sleep in *nca(lf)* mutants that express *nca-1* driven by a panneuronal promoter. As a result, panneuronal expression of *nca-1* almost completely rescued the DTS phenotype of the *nca(lf)* mutants (Fig. 4, a to d, Supplementary Fig. 7 in File 1), indicating that *nca-1* expression in neurons is necessary for DTS regulation. On the other hand, panneuronal expression of *nca-1* did not rescue the SIS phenotype (Fig. 4, e to h, Supplementary Fig. 8 in File 1) of the *nca(lf)* mutants. However, in this experiment, the control *nca(lf)* mutants did not exhibit increased sleep 0.5∼5 h after heat stress in the first place, as observed in Fig. 3, e and g. All animals used in this experiment carried a transgene expressing channelrhodopsin-2 in a subset of interneurons. This transgene might have had unknown effects on the SIS phenotype, which might confound the interpretation of this rescue experiment.

### The *nca-1* gain-of-function mutant exhibits reduced amounts of DTS and SIS

**Fig. 5.**
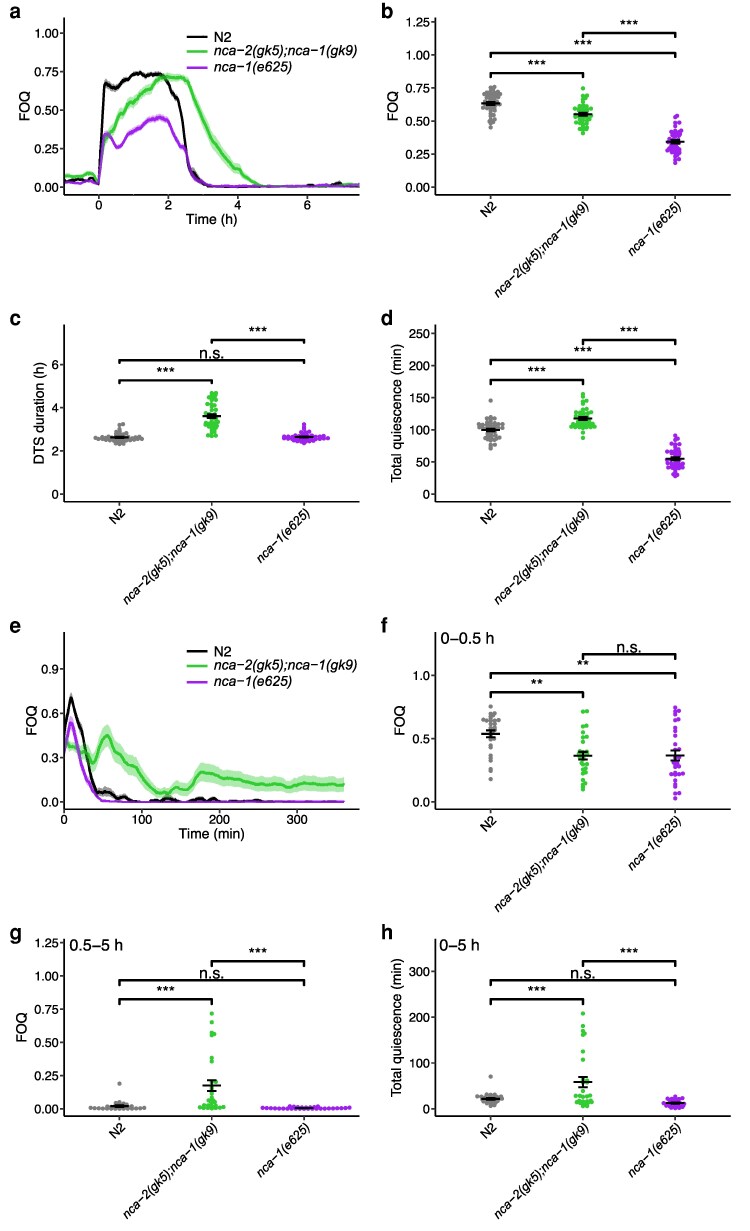
*nca-1(gf)* mutants exhibit lower FOQ during DTS and SIS immediately after heat stress. (a) and (b) Temporal pattern of FOQ (a) and FOQ (b) during DTS for N2 (*n* = 47), *nca-2(gk5); nca-1(gk9)* (*n* = 44), and *nca-1(e625)* (*n* = 44). c) Duration of DTS for N2 (*n* = 47), *nca-2(gk5); nca-1(gk9)* (*n* = 44), and *nca-1(e625)* (*n* = 44). d) Total amount of quiescence during DTS for N2 (*n* = 47), *nca-2(gk5); nca-1(gk9)* (*n* = 44), and *nca-1(e625)* (*n* = 44). e to h) Temporal pattern of FOQ (e), FOQ 0 to 0.5 h (f), FOQ 0.5 to 5 h (g) and total amount of quiescence 0 to 5 h (h) after heat stress exposure at 37 °C for 20 min for N2 (*n* = 30), *nca-2(gk5); nca-1(gk9)* (*n* = 30), and *nca-1(e625)* (*n* = 30). In (a) and (e), the colored lines represent the mean FOQ and shadows around the lines represent SE. In (b to d) and (f to h), each dot represents an individual animal, and bars represent mean ± SE. Multiple comparisons were performed using Tukey-Kramer test. **P < 0.01; ***P < 0.001; n.s., not significant.

*nca-1* gain-of-function (*nca-1(gf)*) mutants are reported to have sleep defects during DTS (Huang et al. 2018). Since the NALCN channel complex regulates membrane potential, precise tuning of its activity within an appropriate dynamic range might be critical for sleep regulation. We therefore hypothesized that, in addition to *nca(lf)* mutants, nca-1(gf) mutants would also exhibit defects in temporal pattern of sleep. To test this hypothesis, we measured DTS and SIS in *nca-1(gf)* mutants. The results showed that *nca-1(gf)* mutants, like *nca(lf)* mutants, exhibited a lower FOQ during DTS and fragmented DTS (Fig. 5, a and b, Supplementary Fig. 9 in File 1). However, they did not show prolonged DTS duration, and the total amount of sleep was less than that of N2 (Fig. 5, c and d). Furthermore, in SIS, *nca-1(gf)* mutants exhibited a lower FOQ immediately after heat stress compared with N2 (Fig. 5, e and f), but no significant sleep fragmentation was observed (Supplementary Fig. 10 in File 1), and there was no subsequent increase in the FOQ (Fig. 5h). These results indicate that *nca-1(gf)* mutants have a low FOQ but do not have the sleep state prolongation seen in *nca(lf)* mutants in both DTS and SIS. These findings suggest that the function of *nca-1* in sleep regulation is complex and that an appropriate level of *nca-1* function is important for sleep stabilization.

### Sleep deprivation immediately after stress results in a sleep pattern similar to that of *unc-80* loss-of-function mutants

**Fig. 6.**
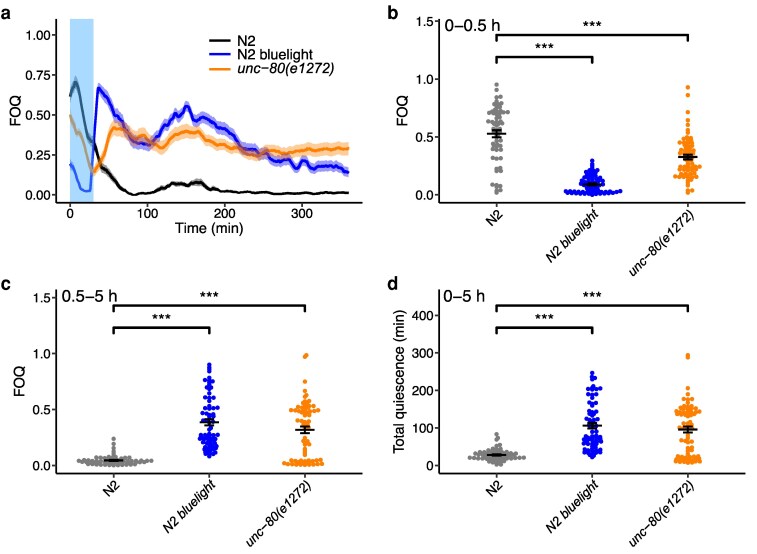
N2 exposed to blue light has similar SIS patterns to *unc-80(e1272)* mutants. a to d) Temporal pattern of FOQ (a), FOQ 0 to 0.5 h (b), 0.5 to 5 h (c) and total amount of quiescence 0 to 5 h (d) after heat stress exposure at 37 °C for 20 min for N2 (*n* = 64), N2 exposed to blue light immediately after heat stress exposure for 30 min (N2 blue light) (*n* = 72), and *unc-80(e1272)* (*n* = 72). In (a), the colored lines represent the mean FOQ and shadows around the lines represent SE. The blue shadow represents the period of blue light. In (b to d), each dot represents an individual animal, and bars represent mean ± SE. Multiple comparisons were performed using Dunnett's test with N2 as a control. ***P < 0.001.

SIS is homeostatically regulated. When worms are deprived of SIS, they show rebound sleep afterward (Soto et al. 2019). *nca(lf)*, *unc-79(lf)*, and *unc-80(lf)* mutants maintained a higher FOQ than N2 for 0.5 to 5 h after heat stress (Fig. 3, e and g). Therefore, we hypothesized that this abnormal timing of sleep may be rebound sleep caused by insufficient sleep immediately after heat stress. To examine this hypothesis, we deprived N2 of sleep for 30 min immediately after heat stress with blue light and examined their sleep. The results showed that N2 exposed to blue light had a lower FOQ during the 30 min after heat stress than normal N2 (Fig. 6, a and b), indicating that their sleep was deprived. After the blue light exposure was stopped, the FOQ of the sleep-deprived N2 appeared to increase sharply and remain higher compared with normal N2 for several hours (Fig. 6a). Actually, the FOQ during 0.5 to 5 h after heat stress was higher, resulting in a higher total amount of quiescence during 5 h after heat stress than normal N2 (Fig. 6, c and d). This sleep pattern was very similar to that of *unc-80(lf)* mutants. In addition, blue light exposure without prior heat stress did not increase subsequent sleep (Supplementary Fig. 11 in File 1), indicating that the FOQ increase after blue light exposure was caused by sleep deprivation, not by stress from blue light exposure itself. These results suggested that *unc-80(lf)* mutants might not be able to get enough sleep immediately after heat stress and thus show rebound sleep afterward. Therefore, sleep homeostasis might be maintained in *unc-80(lf)* mutants. Furthermore, these results also suggest that sleep immediately after heat stress is under strong homeostatic regulation.

### *nca* loss-of-function and *nca-1* gain-of-function mutants have less sleep immediately after severe heat stress and have defects in survival after severe heat stress

**Fig. 7.**
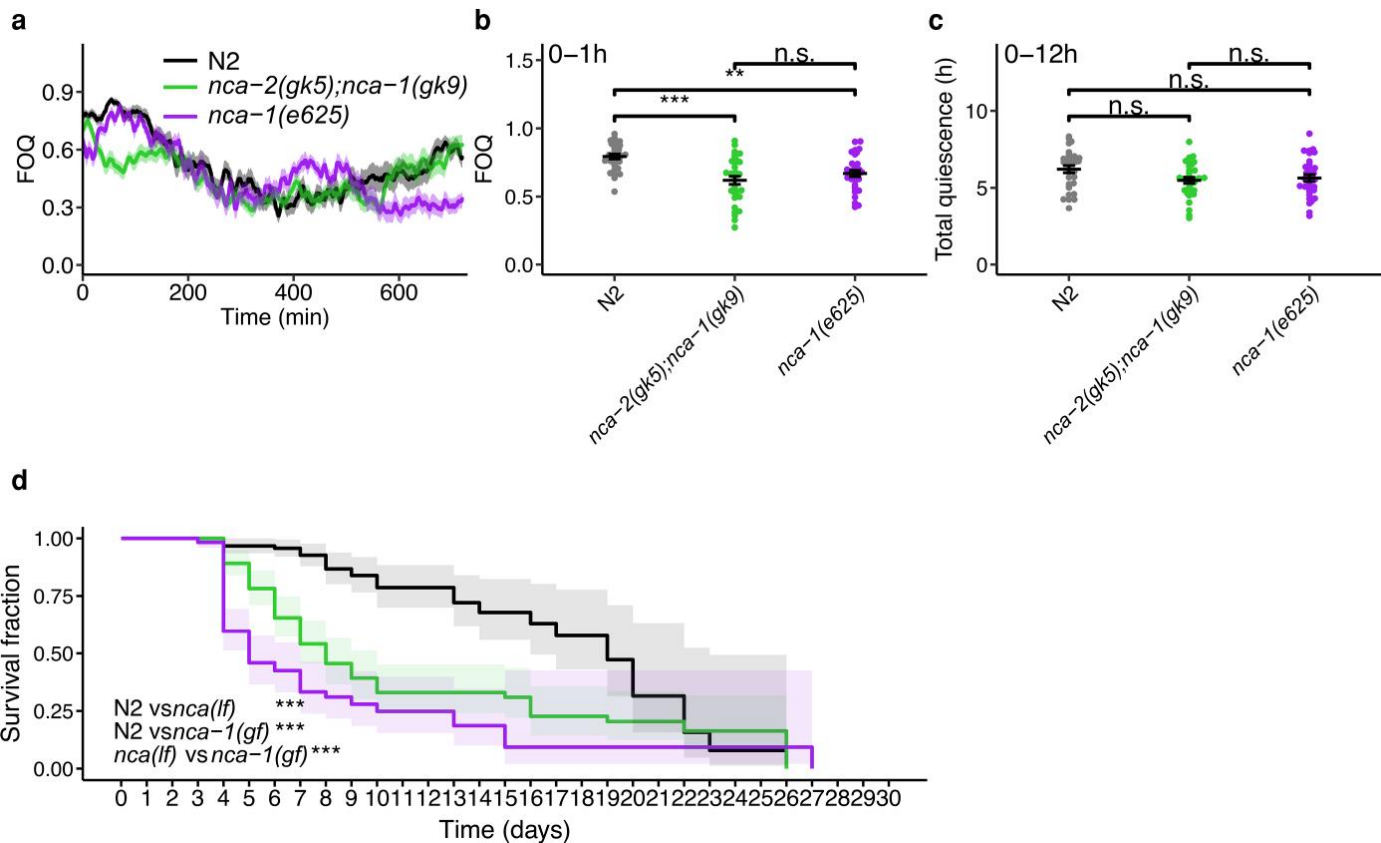
*nca(lf)* and *nca-1(gf)* mutants have defects of SIS and lower survival fraction after severe heat stress. a to c) Temporal patterns of FOQ (a), FOQ 0 to 1 h (b) and total amount of quiescence 0 to 12 h (c) after severe heat stress exposure at 40 °C for 20 min for N2 (*n* = 30), *nca-2(gk5); nca-1(gk9)* (*n* = 32), and *nca-1(e625)* (*n* = 32). d) Survival fraction after severe heat stress exposure at 40 °C for 20 min for N2 (*n* = 147), *nca-2(gk5); nca-1(gk9)* (*n* = 145), and *nca-1(e625)* (*n* = 144). In (a) and (d), the colored lines represent the mean FOQ and fraction of surviving animals, respectively, and shadows around the lines represent SE and 95% confidence interval, respectively. In (b) and (c), each dot represents each animal, and bars represent mean ± SE. In (b) and (c), multiple comparisons were performed using Tukey-Kramer test. In (d), multiple comparisons were performed using the pairwise log-rank test with Holm correction. **P < 0.01; ***P < 0.001; n.s., not significant.

Because mutants of NALCN channel complex appear to be unable to establish stable sleep, we hypothesized that these mutants would not show a higher FOQ state than N2 when exposed to more intense stress that requires prolonged sleep in N2. Therefore, we examined sleep of *unc-79(lf)* and *unc-80(lf)* mutants after severe heat stress at 40 °C for 20 min, which subsequently causes long sleep in N2 (Hill et al. 2014). While the FOQ of *unc-79(lf)* mutants was not significantly different from N2, *unc-80(lf)* mutants showed a lower FOQ than N2 (Supplementary Fig. 12, a to c in File 1). We hypothesized that *unc-80(lf)* mutants, which have less sleep, would have lower survival fraction after severe heat stress. However, *unc-80(lf)* mutants showed longer survival after heat stress at 40 °C (Supplementary Fig. 12d in File 1). This mutant strain has longer lifespan, which is suggested to result from unknown side mutations caused by EMS mutagenesis (Lakowski and Hekimi 1998). This life-extending effect may influence the result of the survival experiment. Thus, we examined SIS and survival fraction of *nca(lf)* and *nca-1(gf)* mutants, which also have defects in SIS after heat stress at 37 °C (Fig. 3, e to h and 5, e to h). Both mutants showed a lower FOQ immediately after heat stress compared with N2 (Fig. 7, a and b), which is similar phenotype to that of SIS after heat stress at 37 °C (Figs. 3, e, f and 5, e and f). Although the amount of total quiescence was not significantly different from N2, their survival fractions were lower than that of N2 (Fig. 7, c and d). This result suggests that sleep immediately after heat stress might be especially important for survival.

## Discussion

This study revealed common functions of *nca*, *unc-*79, and *unc-80*, which form the NALCN channelosome (Kschonsak et al. 2022), in sleep stabilization. *nca(lf)*, *unc-79(lf)*, and *unc-80(lf)* mutant *C. elegans* all exhibited a lower FOQ during DTS, and their sleep was fragmented. Despite this unstable sleep phenotype, the duration of their DTS was longer, and the total amount of quiescence was higher than that in N2, consistent with a previous study reporting a similar sleep phenotype in the *unc-79(lf)* mutant DTS (Huang et al. 2018). In contrast to our results, the previous study showed that *nca(lf)* and *unc-80(lf)* mutants had both a normal DTS duration and total amount of quiescence. In that study, each chamber contained numerous small posts mimicking artificial dirt (Huang et al. 2018), but the chamber used in the present study did not have such posts. Worms crawl when such structures are present (Lockery et al. 2008) but swim in their absence. Therefore, the difference in the results may be partly due to distinct crawling and swimming movements observed in the previous and the present study, respectively. In addition, images were acquired every 10 s in the previous study, whereas they were acquired every 2 s in the present study. Therefore, movements of the worms may have been detected more sensitively in the present study, which may have allowed the detection of DTS defects in the mutants. Similarly to DTS, during SIS, *nca(lf)*, *unc-79(lf)*, and *unc-80 (lf)* mutants had a lower FOQ and fragmented sleep for 30 min immediately after mild heat stress (37 °C, 20 min) but then had a higher FOQ for a longer period than N2. This sleep pattern is similar to that of DTS, suggesting that *nca*, *unc-79*, and *unc-80* have similar functions in DTS and SIS regulation. That these mutants showed similar sleep patterns is also consistent with previous studies suggesting that *nca*, *unc-79*, and *unc-80* function in the same pathway (Humphrey et al. 2007; Jospin et al. 2007; Yeh et al. 2008; Speca et al. 2010). In addition, considering frequent cessation of locomotion, a phenotype called “fainter” (Humphrey et al. 2007), and sleep fragmentation of the loss-of-function mutants of these genes, they might exhibit general impairment in maintaining behavioral states. NALCN channel complex regulates resting membrane potential and neuronal excitability (Ren 2011) and has been suggested to function upstream of gap junction proteins (Huang et al. 2018). Therefore, these mutants may exhibit inappropriate activity patterns or altered connectivity in neuronal circuits required to maintain specific behavioral states. In the present study, *unc-80(lf)* mutants showed normal arousal thresholds during SIS, while the previous study showed that *nca(lf)*, *unc-79(lf)* and *unc-80(lf)* mutants have higher arousal thresholds during sleep bouts in DTS (Huang et al. 2018). Based on these results, *nca*, *unc-79*, and *unc-80* might be involved in regulating the arousal threshold only during DTS.

*nca-1* and *nca-2* function redundantly for various phenotypes (Yeh et al. 2008; Huang et al. 2018). Furthermore, *nca-1* is expressed in many neurons, including motor neurons, interneurons, and sensory neurons (Jospin et al. 2007; Yeh et al. 2008). Panneuronal expression of *nca-1* rescued DTS defects of *nca(lf)* mutants, which is consistent with these findings, but failed to rescue SIS defects. These results suggest that *nca-1* functions redundantly with *nca-2* within neurons in the regulation of DTS, but not SIS. However, these results should be interpreted with caution because the strain we used for *nca(lf)* mutant rescue carried a genome-integrated transgene *hpIS166[Pglr-1::ChR2::YFP]* (Gao et al. 2015), which might affect the results of the rescue experiment. In the future, rescuing *nca-1* in different subsets of neurons would help elucidate neural mechanisms by which NALCN channel complex regulates sleep in greater detail.

*nca-1(gf)* mutants exhibited a lower FOQ during DTS and during SIS immediately after heat stress, similar to *nca(lf)* mutants. *nca(lf)* and *nca-1(gf)* mutants exhibit decreased and increased calcium activity at presynaptic terminals, respectively (Yeh et al. 2008). This suggests that in *nca(lf)* mutants, the FOQ decreased due to reduced activity in neurons involved in sleep regulation, while in *nca-1(gf)* mutants, the FOQ decreased due to increased activity in motor neurons. At the organismal level, this indicates that an appropriate level of functioning of the NALCN channel complex is crucial for stabilizing sleep.

One of the behavioral definitions of sleep is homeostatic regulation, i.e. the occurrence of rebound sleep after sleep deprivation (Campbell and Tobler 1984; Rial et al. 2010). N2 animals deprived of sleep for 30 min immediately after heat stress remained in a high FOQ state for a long period after sleep deprivation, whereas N2 animals without sleep deprivation exhibited a high FOQ immediately after heat stress and then the FOQ quickly decreased. This sleep pattern of sleep-deprived N2 animals was very similar to that of the *nca(lf)*, *unc-79(lf)*, and *unc-80(lf)* mutants. These results suggest that the long-lasting high FOQ state observed in these mutants is a secondary result of insufficient sleep immediately after heat stress, similar to the rebound sleep in N2 caused by sleep deprivation, which further suggests that sleep homeostasis is at least partly retained in these mutants. Moreover, in *nca(lf)*, *unc-79(lf)*, and *unc-80(lf)* mutants, and sleep-deprived N2, the total quiescence was considerably greater than that in non-sleep-deprived N2. Perhaps the early period of post-stress sleep is essential and thus is under strong homeostatic regulation. In addition to SIS, these mutants appeared to have a lower FOQ, especially in the first half of DTS, and their total quiescence was higher than that in N2. This sleep pattern might suggest that, like SIS, the early period of DTS is also under strong homeostatic regulation and essential. Lack of sleep induces a proteostatic disruption as assessed by the increased expression of chaperones (Cirelli et al. 2006; Anafi et al. 2013; Hill et al. 2014; Naidoo et al. 2014; Sanders et al. 2017). Therefore, if the early period of sleep is particularly important, such sleep is expected to be essential for the expression of these proteins and the maintenance of proteostasis. In this case, losing sleep during the early period of sleep may lead to a greater subsequent need for sleep than the amount initially lost, as observed following mild heat stress, to maintain sleep homeostasis. Comparing the expression levels of these proteins during the early and later periods of sleep may reveal the particular importance of the early period of sleep. After severe heat stress (40 °C, 20 min), *unc-80(lf)* mutants exhibited a markedly lower FOQ immediately afterward and continued to maintain a slightly lower FOQ compared with N2, without displaying a rebound sleep-like state. This may be due to the fact that after severe heat stress, even N2 requires a high FOQ for a long time, and thus the FOQ of *unc-80(lf)* mutants, which cannot stabilize sleep, could not exceed the FOQ of N2. After severe heat stress, *nca(lf)* and *nca-1(gf)* mutants, both of which had lower sleep amount immediately after heat stress, showed lower survival fraction. Considering that their total sleep amount was not significantly different from N2, there is a possibility that sleep immediately after onset plays an important role for survival. In humans, sleep immediately after onset is suggested to be especially important for health (VanCauter and Plat 1996; Besedovsky et al. 2012; Javaheri et al. 2017; Himali et al. 2023). Therefore, this particular importance of sleep immediately after sleep onset might be conserved widely among animal species.

There are several limitations to our study. How mutations in *nca-1, nca-2*, *unc-79*, and *unc-80* lead to sleep disruption remain unclear. In the future, it is crucial to test how the activity of sleep-regulating neurons, such as RIS (Turek et al. 2013; Kucherenko et al. 2016; Hu et al. 2020; Nakai et al. 2020) or ALA (Hill et al. 2014), are affected in *nca*, *unc-79*, and *unc-80* mutants. In addition, it is also important to test the genetic interactions between *nca*, *unc-79*, or *unc-80* and genes that are essential for differentiation of the sleep-regulating neurons and to conduct cell-specific rescue experiments of the *nca*, *unc-79*, and *unc-80* mutants.

*NALCN*, *UNC79*, and *UNC80* are widely conserved in the animal kingdom. Mutations in these genes cause sleep defects in flies (Murakami et al. 2021), mice (Funato et al. 2016), and humans (Stray-Pedersen et al. 2016; Angius et al. 2018; He et al. 2018). We found that that these genes are also involved in sleep stabilization, both in DTS and SIS, in *C. elegans*. To determine whether this function is conserved across the animal kingdom, further studies are needed to investigate detailed sleep patterns and total sleep amounts in mutants of these genes in other animals.

## Supplementary Material

jkag112_Supplementary_Data

## Data Availability

All *C. elegans* strains used in this study except for SLP769 are available from the Caenorhabditis Genetics Center. The strains used in this study are available upon request. The python codes used in this study are available at the github repositories (https://github.com/TakahiroKamijo/ImageSubt_analysis.git; https://github.com/TakahiroKamijo/worm_tracking.git) and licensed under the GNU General Public License version 2 (GPL-2.0), which requires that any modifications or derivative works must also be distributed under the same license. All raw data and results of statistical analyses are included in File S2. Supplemental material available at G3 online.
