# Supplementary material for: The NALCN channel complex is necessary for stabilizing sleep in Caenorhabditis elegans
Source: G3 (Bethesda). 2026 May 5;16(7):jkag112. doi: 10.1093/g3journal/jkag112 (PMC13334172; doi:10.1093/g3journal/jkag112)
Supplement: jkag112_Supplementary_Data [file jkag112_supplementary_data.zip › File_S1_G3-2026-406727.docx]

**Supplementary Figures**


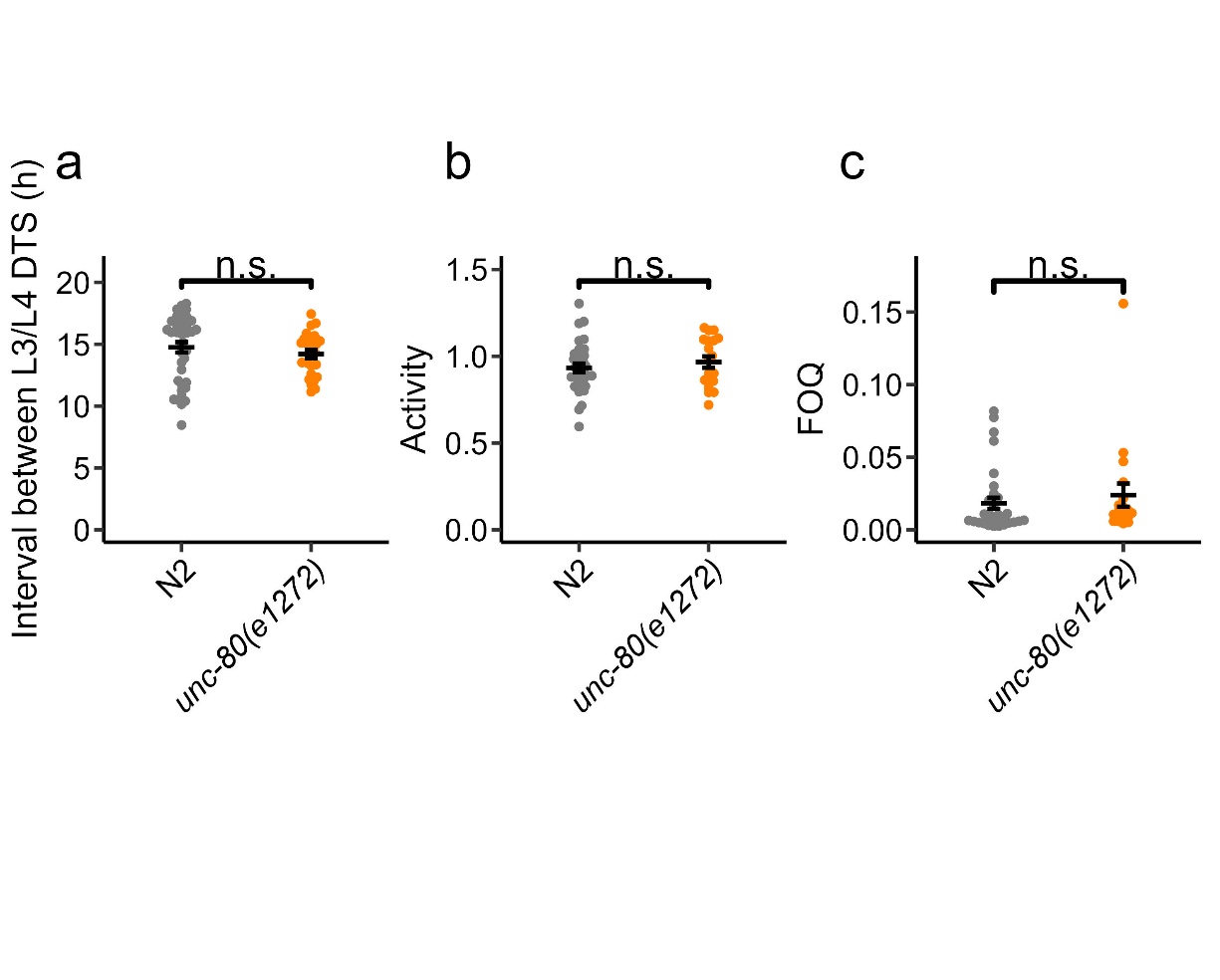


**Fig. S1.** Develomental speed, activity and FOQ out of DTS are normal in *unc-80(lf)* mutants. a-b) Interval between L3/L4 DTS for N2 (n = 41) and *unc-80(e1272)* (n = 26), activity (a) and FOQ (b) out of DTS for N2 (n = 33) and *unc-80(e1272)* (n = 19). Each dot represents each animal, and bars represent mean ± SE. Welch’s t test was performed. n.s., not significant.


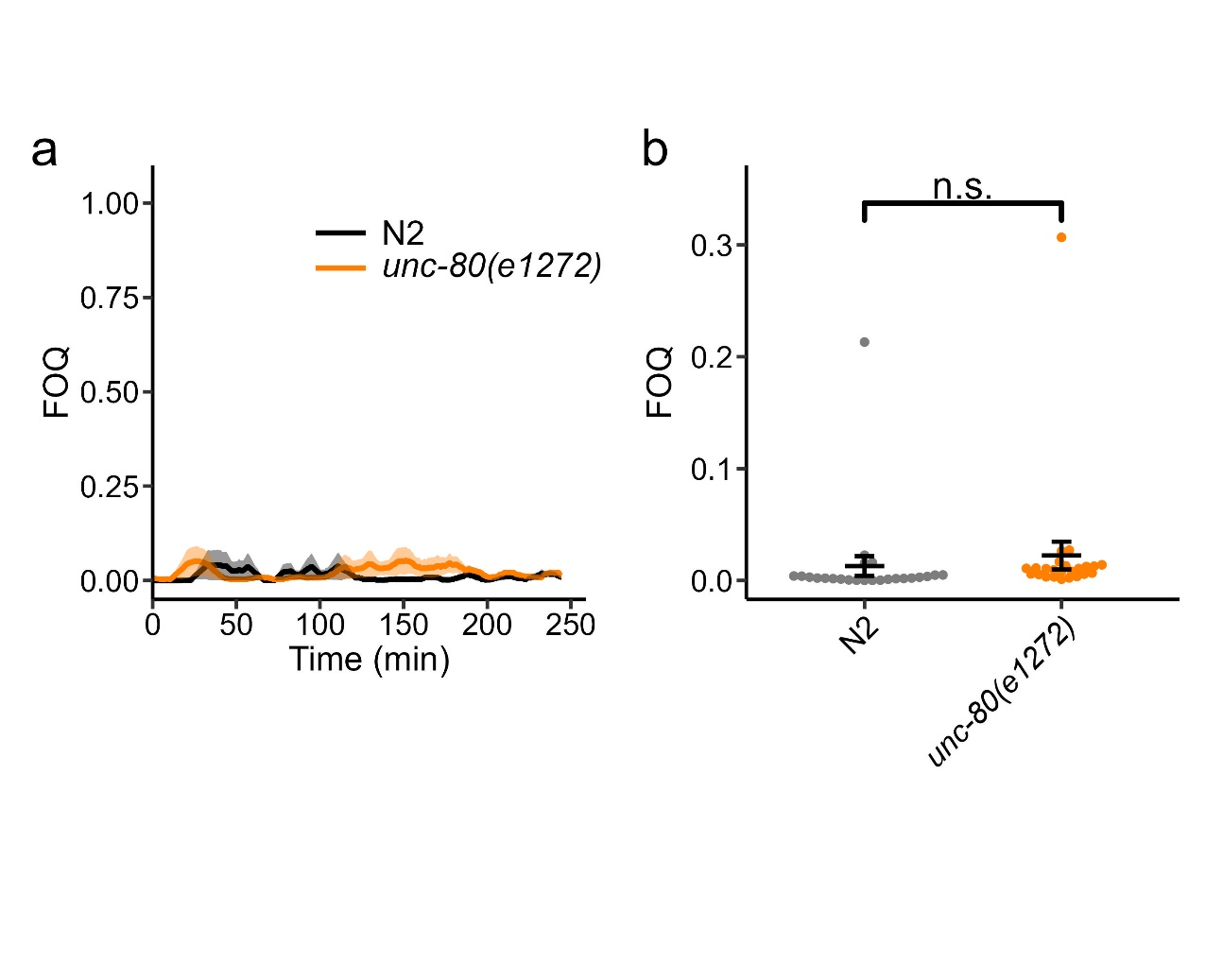


**Fig. S2.** FOQ is normal in *unc-80(e1272)* mutants without heat stress. a-b) Temporal pattern of FOQ (a) and mean FOQ (b) for 4 h for N2 (n = 24) and *unc-80(e1272)* (n = 24). In (a), the colored lines represent the mean FOQ and shadows around the lines represent SE. In (b), each dot represents each animal, and bars represent mean ± SE. Welch’s t test was performed. n.s., not significant.


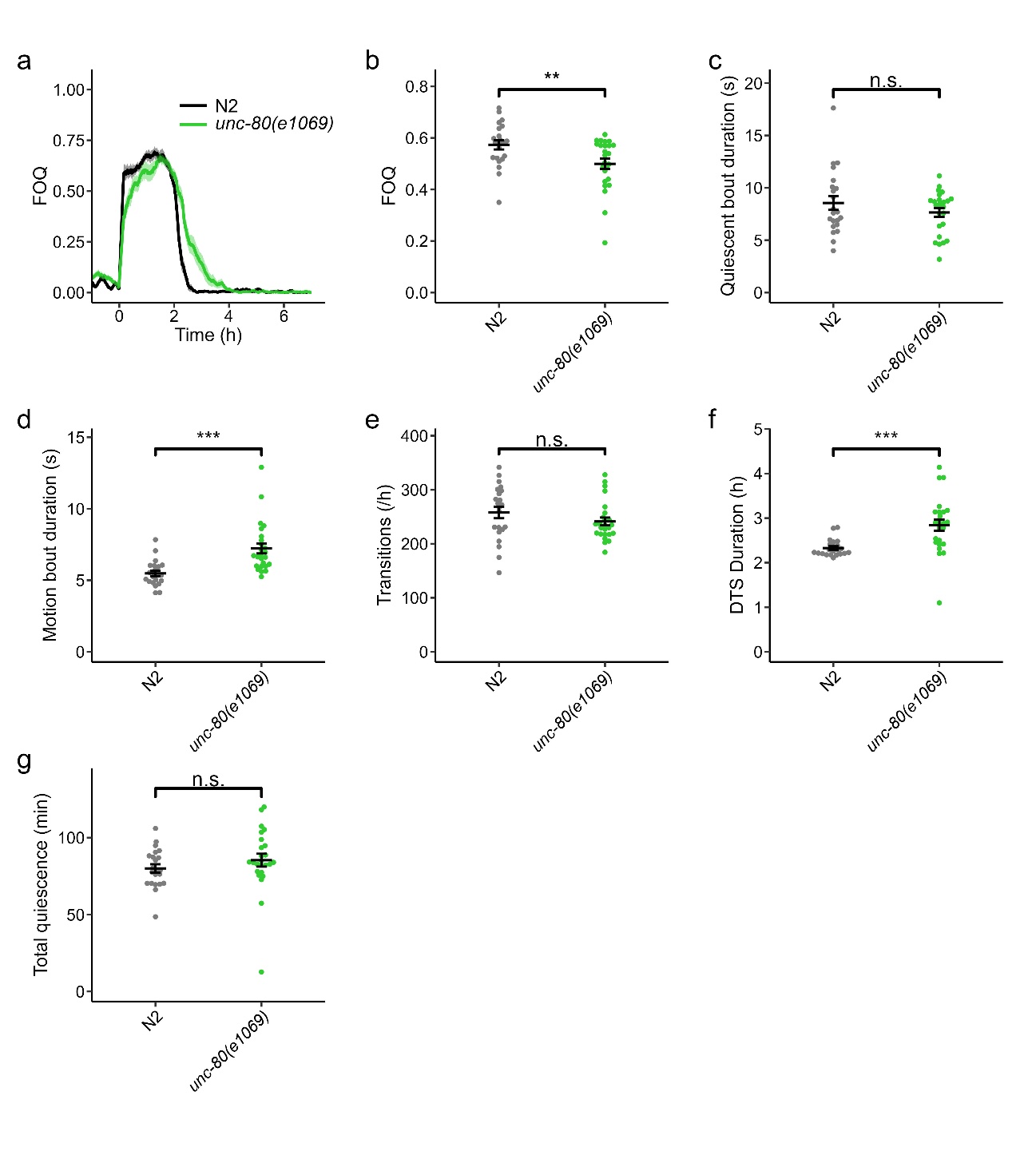


**Fig. S3.** *unc-80(e1069)* mutants have similar DTS features to *unc-80(e1272)* mutants. a-e) Temporal pattern of FOQ (a) and mean FOQ (b), mean duration of quiescent bouts (c), mean duration of motion bouts (d) and transitions between quiescent and motion bouts per hour (e) during DTS for N2 (n = 22) and *unc-80(e1069)* (n = 25). f) Duration of DTS for N2 (n = 22) and *unc-80(e1069)* (n = 25). g) Total amount of quiescent state during DTS for N2 (n = 22) and *unc-80(e1069)* (n = 25). In (a), the colored lines represent the mean FOQ and shadows around the lines represent SE. In (b)-(g), each dot represents each animal, and bars represent mean ± SE. Welch’s t test was performed. **P<0.01; ***P<0.001; n.s., not significant.


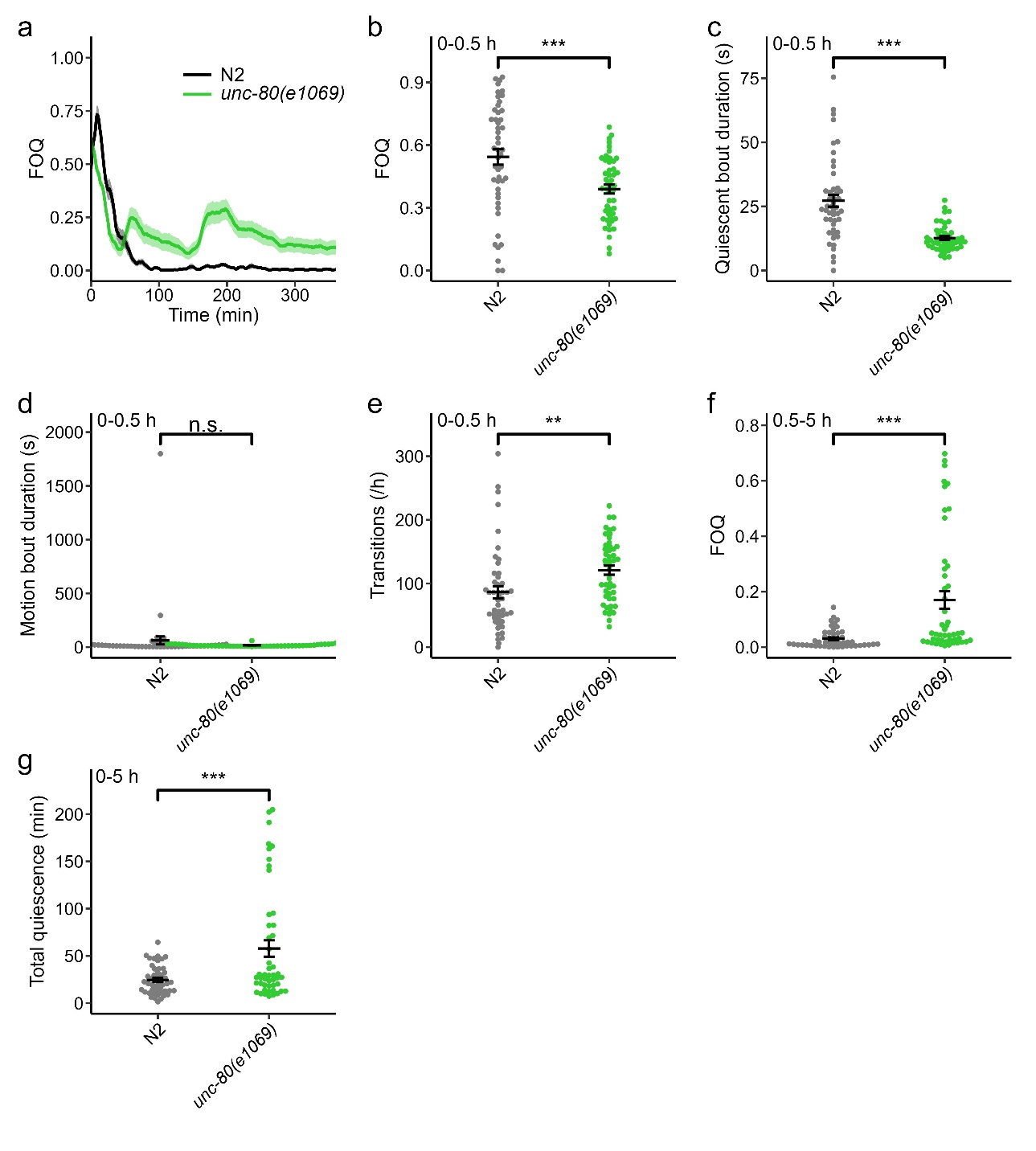


**Fig. S4.** *unc-80(e1069)* mutants have similar SIS features to *unc-80(e1272)* mutants. a) Temporal pattern of FOQ . b-d) FOQ (b), mean duration of quiescent bouts (c), mead duration of motion bouts (d) and transitions between quiescent and motion bouts per hour (e) during 0~0.5 h after heat stress for N2 (n = 48) and *unc-80(e1272)* (n = 48). f) FOQ during 0.5-5 h after heat stress for N2 (n = 48) and *unc-80(e1272)* (n = 48). g) total amount of quiescence during 0~5 h after heat stress for N2 (n = 48) and *unc-80(e1272)* (n = 48). In (a), the colored lines represent the mean FOQ and shadows around the lines represent SE. In (b)-(g), each dot represents each animal, and bars represent mean ± SE. Welch’s t test was performed. **P<0.01; ***P<0.001; n.s., not significant.


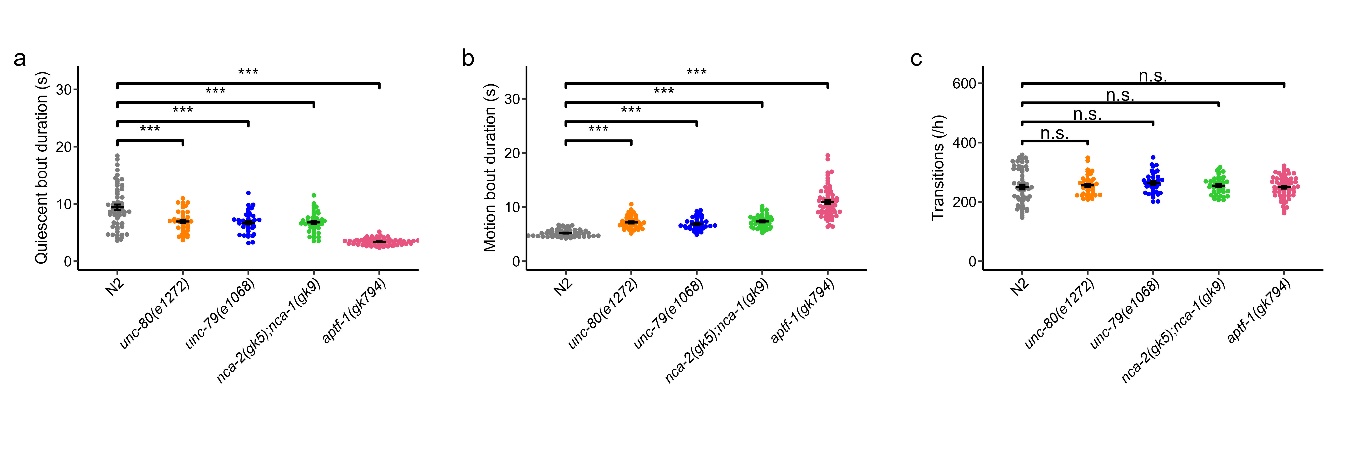
**Fig. S5.** *unc-79(e1068)* mutants and *nca-2(gk5); nca-1(gk9)* double mutants have similar DTS features to *unc-80(e1272)* mutants. a-c) Mean duration of quiescent bouts (a), mean duration of motion bouts (b) and transitions between quiescent and motion bouts per hour (c) during DTS for N2 (n = 58), *unc-80(e1272)* (n = 40), *unc-79(e1068)* (n = 37), *nca-2(gk5); nca-1(gk9)* (n = 42), and *aptf-1(gk794)* (n = 63). Each dot represents each animal, and bars represent mean ± SE. Multiple comparisons were performed using Dunnett’s test with N2 as control. ***P<0.001; n.s., not significant.


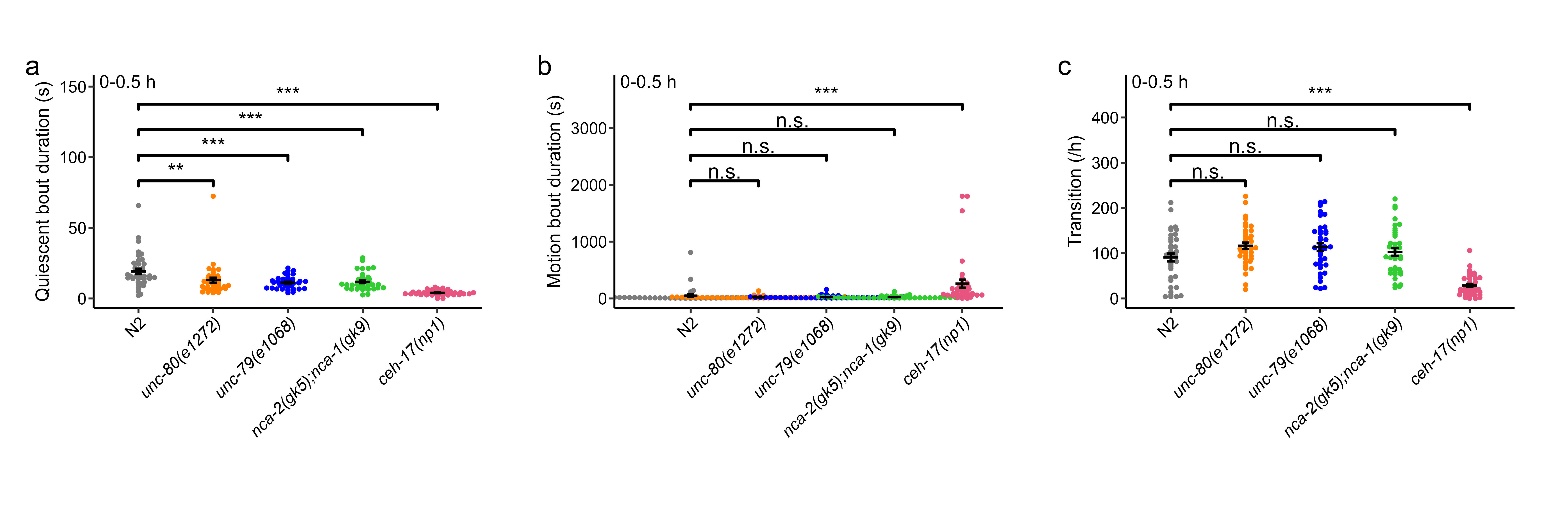


**Fig. S6.** *unc-79(e1068)* mutants and *nca-2(gk5); nca-1(gk9)* double mutants have similar SIS features to *unc-80(e1272)*. a-c) Mean duration of quiescent bouts (a), mean duration of motion bouts (b) and transitions between quiescent and motion bouts per hour (c) during 0~0.5 h after heat stress for N2 (n = 39), *unc-80(e1272)* (n = 39), *unc-79(e1068)* (n = 39), *nca-2(gk5); nca-1(gk9)* (n = 39), and *ceh-17(np1)* (n = 39). Each dot represents each animal, and bars represent mean ± SE. Multiple comparisons were performed using Dunnett’s test with N2 as control. **P<0.01; ***P<0.001; n.s., not significant.


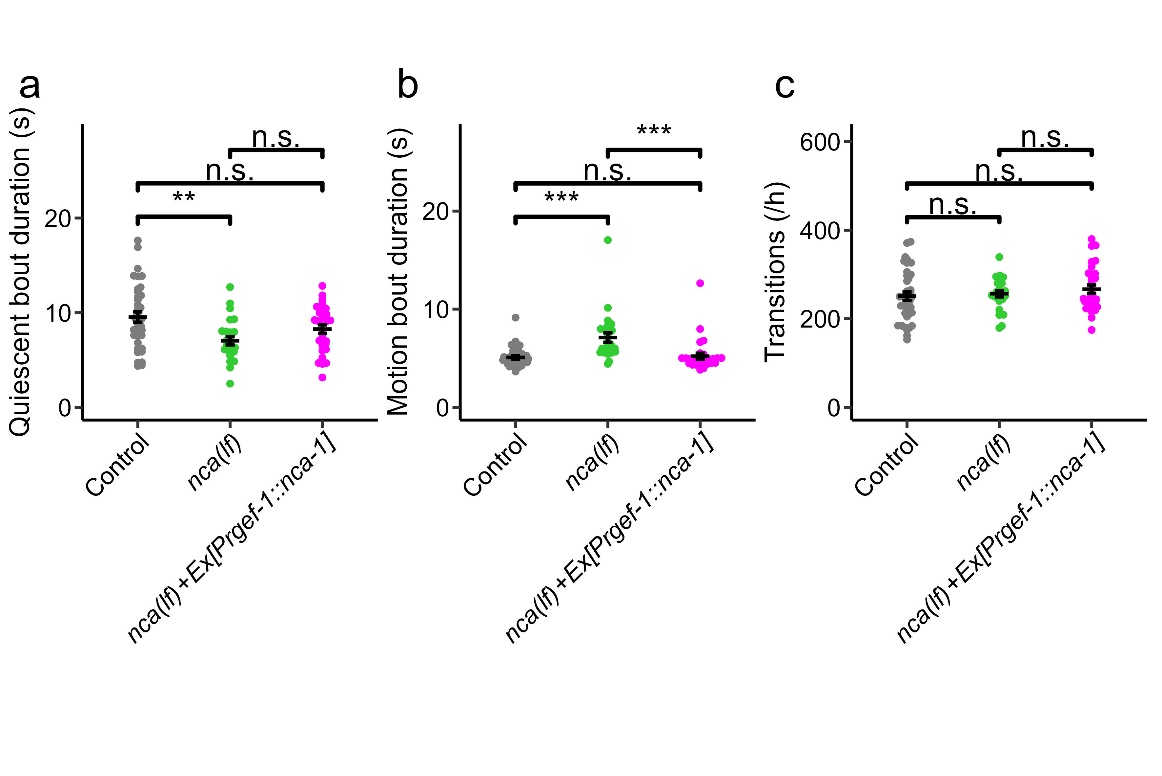


**Fig. S7.** Panneuronal expression of *nca-1* rescues the defect of motion bout duration during DTS of the *nca-2(gk5); nca-1(gk9)* double mutants. a-c) Mean duration of quiescent bouts (a), mean duration of motion bouts (b) and transitions between quiescent and motion bouts per hour (c) during DTS for control (*Pglr-1::ChR2::YFP*) (n = 38), *nca-2(gk5); nca-1(gk9); Pglr-1::ChR2::YFP* (n = 26), and *nca-2(gk5); nca-1(gk9); Pglr-1::ChR2::YFP; Prgef-1::nca-1::GFP*(n = 30). Each dot represents each animal, and bars represent mean ± SE. Multiple comparisons were performed using Tukey-Kramer test. **p<0.01; ***p<0.001; n.s., not significant.


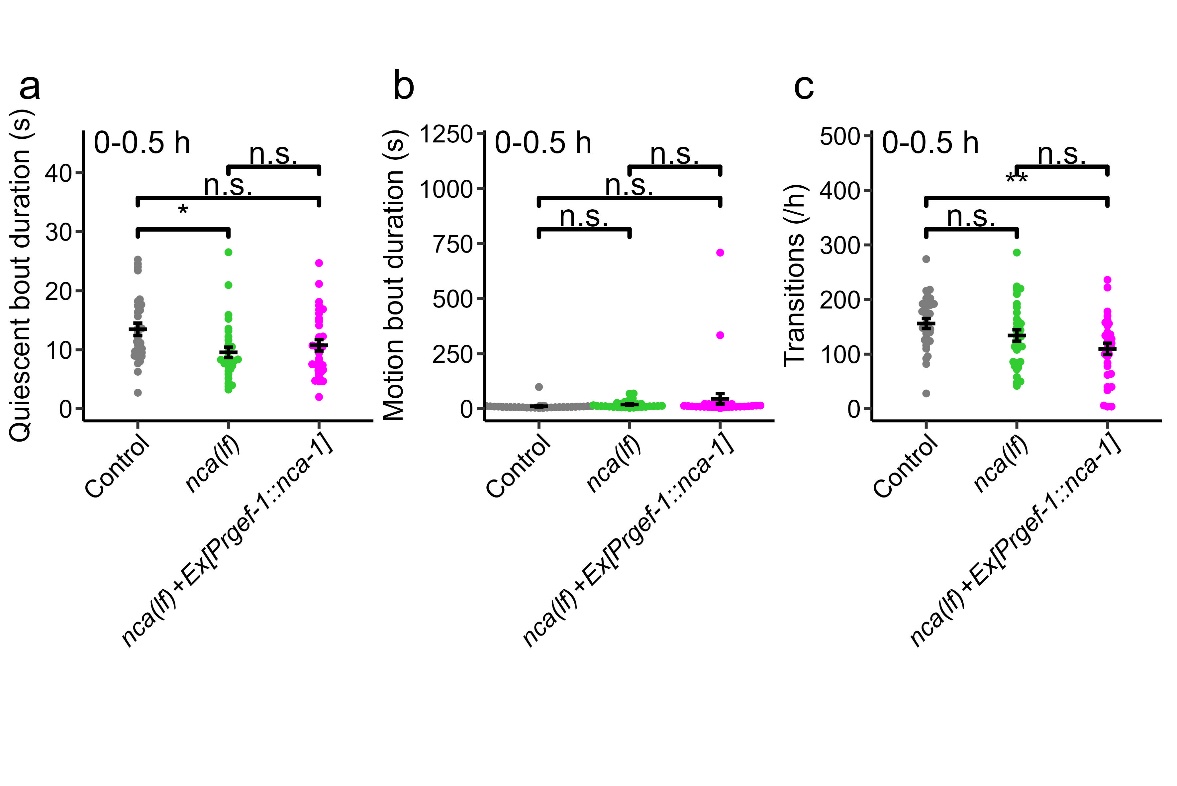


**Fig. S8.** Panneuronal expression of *nca-1* does not rescue SIS defects of the *nca-2(gk5); nca-1(gk9)* double mutants. a-c) Mean duration of quiescent bouts (a), mean duration of motion bouts (b) and transitions between quiescent and motion bouts per hour (c) during 0~0.5 h after heat stress for control (*Pglr-1::ChR2::YFP*) (n = 30), *nca-2(gk5); nca-1(gk9); Pglr-1::ChR2::YFP* (n = 32), and *nca-2(gk5); nca-1(gk9); Pglr-1::ChR2::YFP; Prgef-1::nca-1::GFP*(n = 32). Each dot represents each animal, and bars represent mean ± SE. Multiple comparisons were performed using Tukey-Kramer test. *p<0.05; **p<0.01; n.s., not significant.


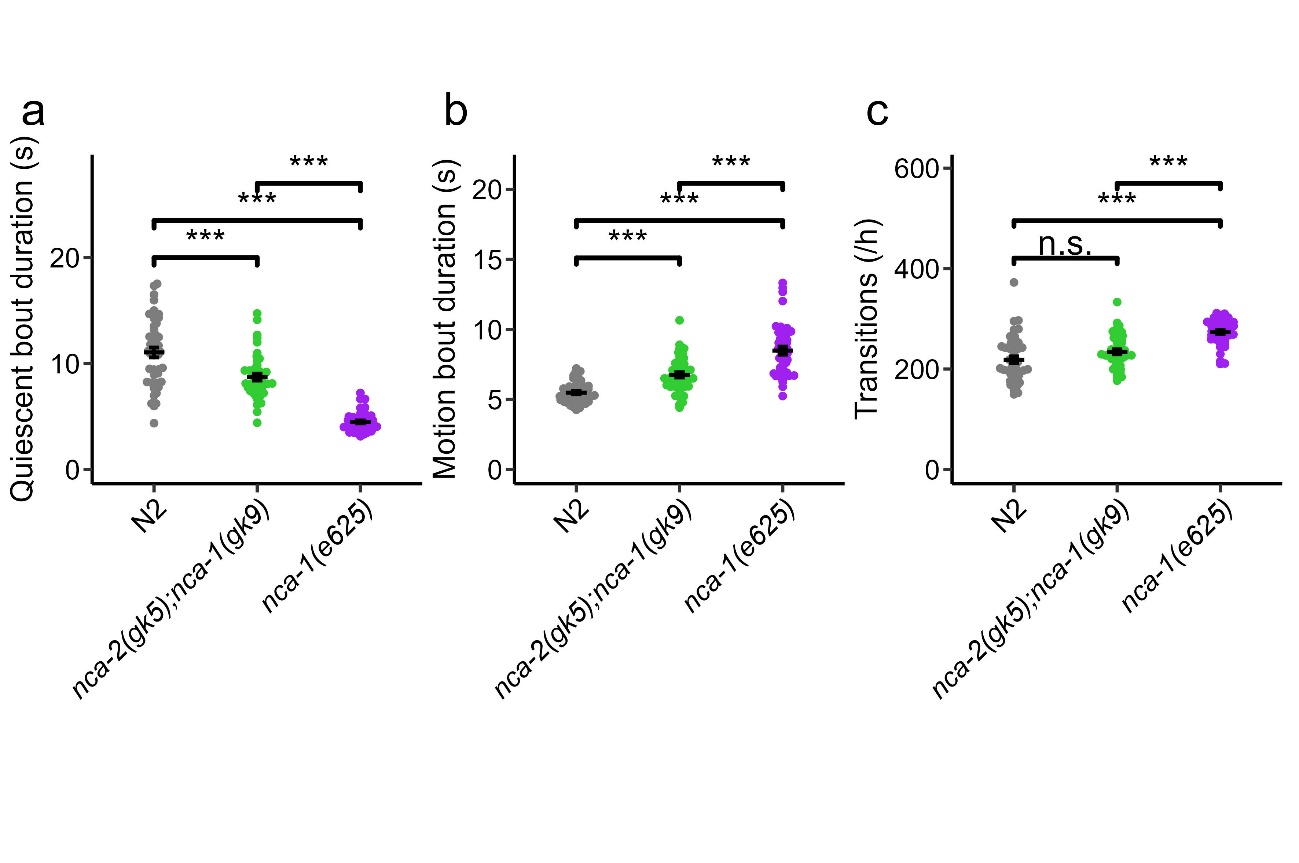


**Fig. S9.** DTS of *nca-1(gf)* mutants is fragmented. a-c) Mean duration of quiescent bouts (a), mean duration of motion bouts (b) and transitions between quiescent and motion bouts per hour (c) during DTS for N2 (n = 47), *nca-2(gk5); nca-1(gk9)* (n = 44), and *nca-1(e625)* mutants (n = 44). Each dot represents each animal, and bars represent mean ± SE. Multiple comparisons were performed using Tukey-Kramer test. ***p<0.001; n.s., not significant.


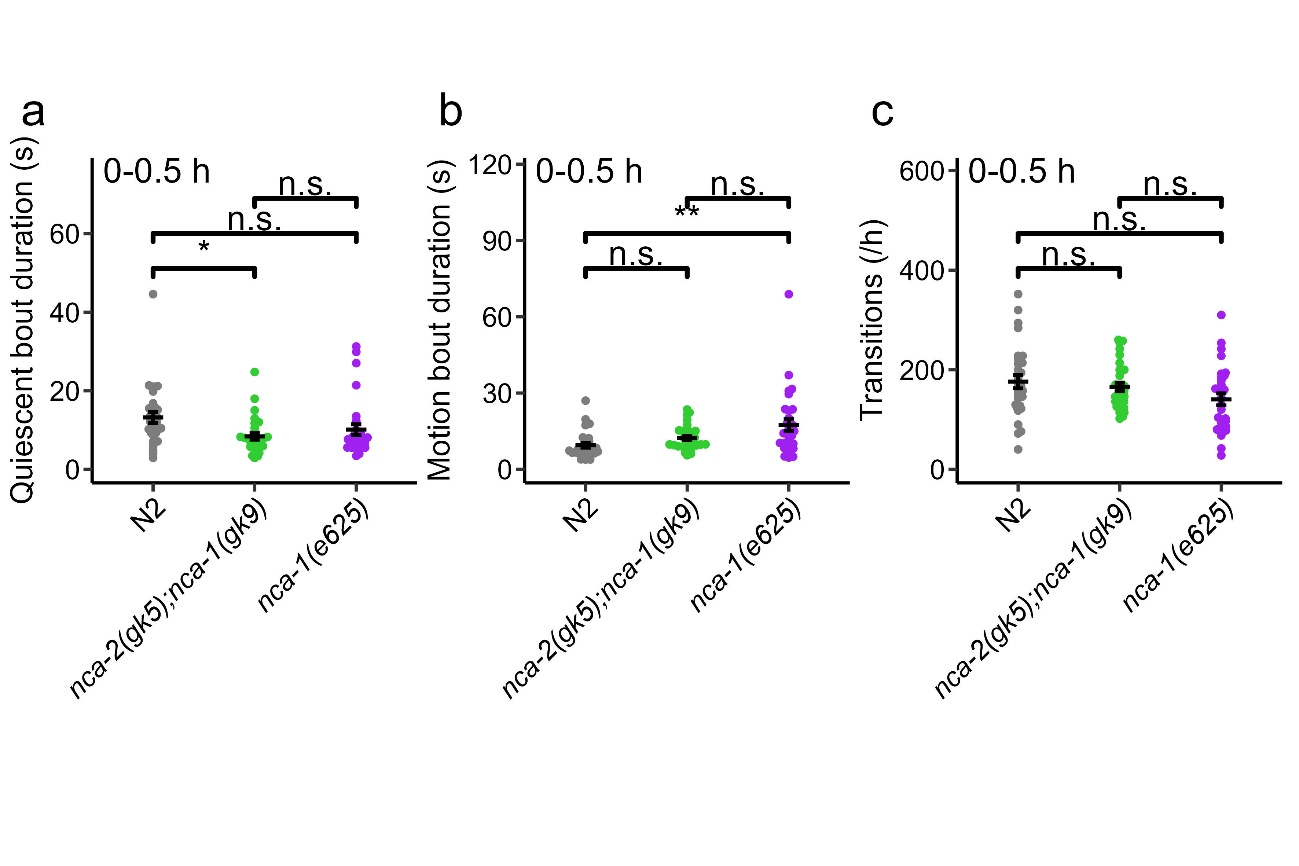


**Fig. S10.** SIS of *nca-1(gf)* mutants is not fragmented. a-c) Mean duration of quiescent bouts (a), mean duration of motion bouts (b) and transitions between quiescent and motion bouts per hour (c) during 0~0.5 h after heat stress for N2 (n = 30), *nca-2(gk5); nca-1(gk9)* (n = 30), and *nca-1(e625)* mutants (n = 30). Each dot represents each animal, and bars represent mean ± SE. Multiple comparisons were performed using Tukey-Kramer test. *p<0.05; **p<0.01; n.s., not significant.


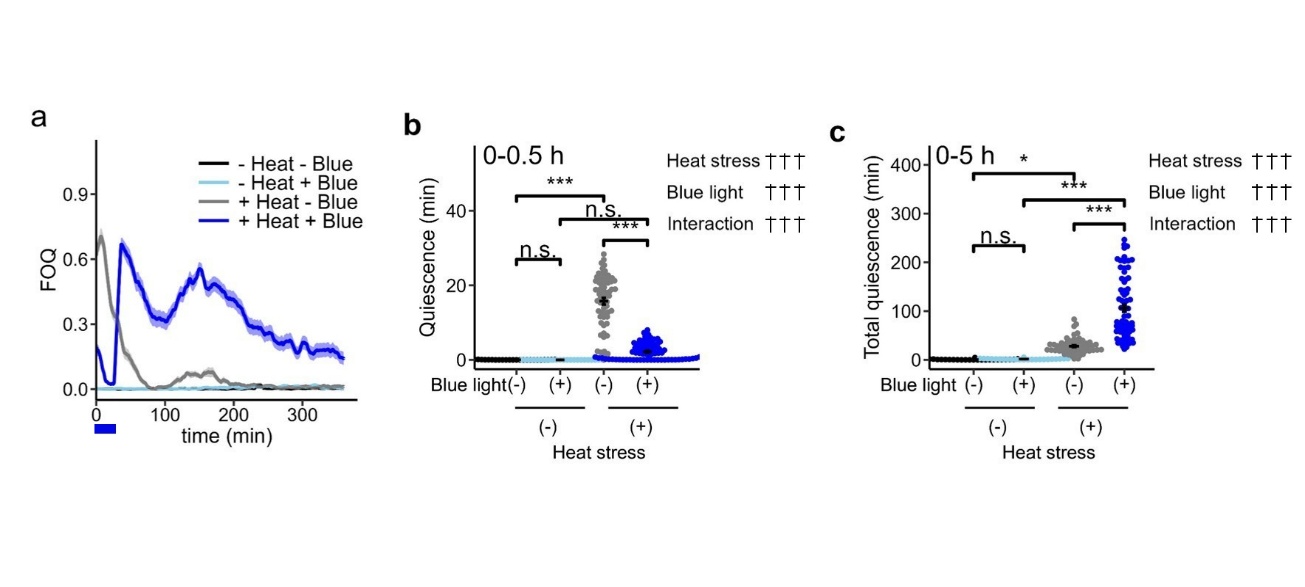
**Fig. S11.** Blue light exposure increases subsequent sleep only when combined with heat stress. a-d) Temporal patterns of FOQ (a), amount of quiescence during 0-0.5 h (b) and total amount of quiescence 0-5 h (c) for no heat stress/no blue light (n = 24), blue light alone(n = 24), heat stress alone (n = 64) (the same data from Fig. 6) and heat stress/blue light (n = 72) (the same data from Fig. 6). In (a), the colored lines represent the mean FOQ and shadows around the lines represent SE. In (b) and (c), each dot represents each animal, and bars represent mean ± SE. In (b) and (c), a dagger symbol (†) indicates significance in two-way ANOVA. †††p<0.001. Multiple comparisons were performed using post hoc Tukey-Kramer test. *p<0.05; ***p<0.001; n.s., not significant.


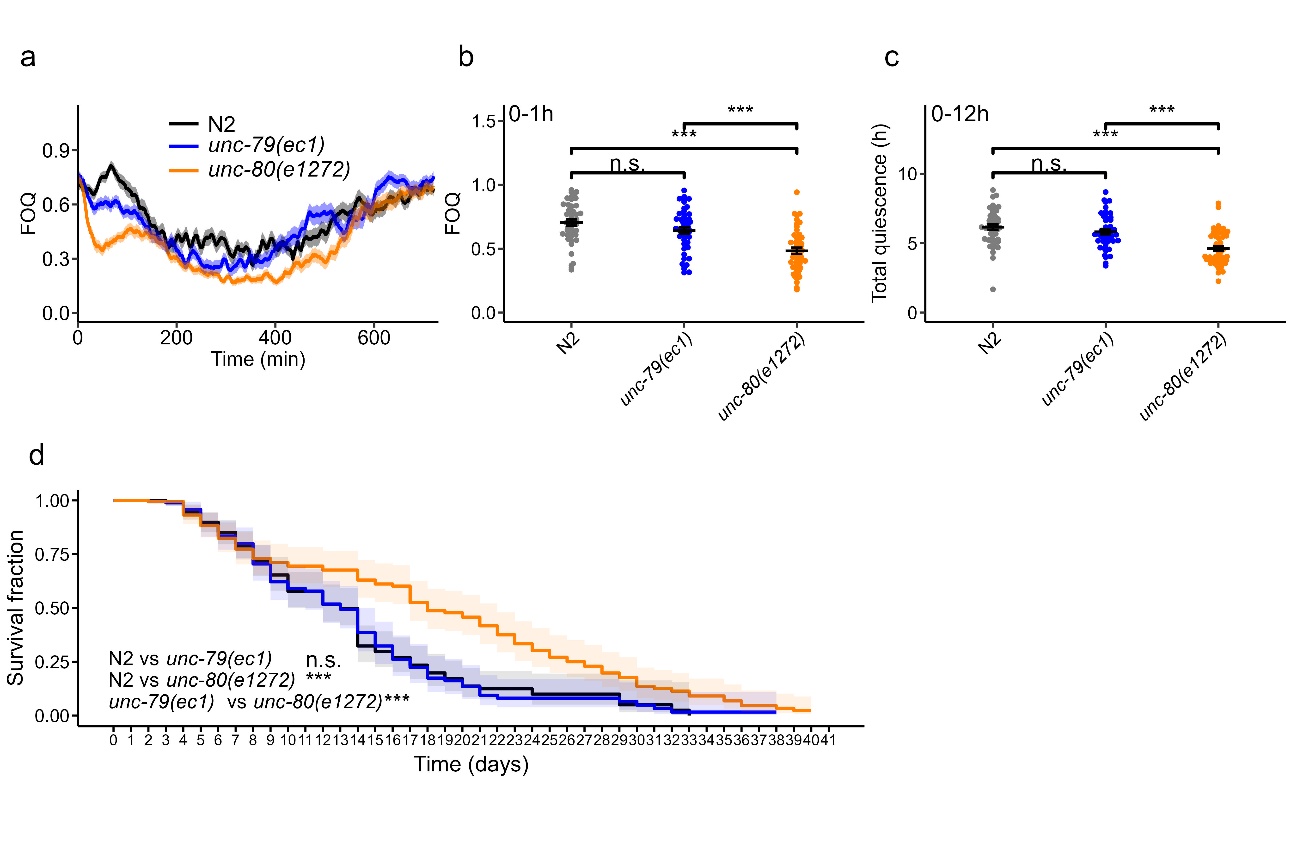


**Fig. S12.** *unc-80(e1272)* mutants exhibit SIS defects but their survival fraction after severe heat stress is not reduced. a-d) Temporal patterns of FOQ (a), FOQ 0-1 h (b) and total amount of quiescence 0-12 h (c) after severe heat stress exposure at 40°C for 20 min for N2 (n = 45), *unc-79(ec1)* (n = 47), and *unc-80(e1272)* (n = 48). d)Survival fraction after severe heat stress exposure at 40°C for 20 min for N2 (n = 216), *unc-79(ec1)* (n = 217), and *unc-80(e1272)* (n = 216). In (a) and (d), the colored lines represent the mean FOQ and fraction of surviving animals, respectively, and shadows around the lines represent SE. In (b) and (c), each dot represents each animal, and bars represent mean ± SE. In (b) and (c), multiple comparisons were performed using Welch’s t test with Holm correction. In (d), multiple comparisons were performed using the pairwise log-rank test with Holm correction. *p<0.05; ***p<0.001; n.s., not significant.
